# HIV-1 Uncoating and Reverse Transcription Require eEF1A Binding to Surface-Exposed Acidic Residues of the Reverse Transcriptase Thumb Domain

**DOI:** 10.1128/mBio.00316-18

**Published:** 2018-03-27

**Authors:** Daniel J. Rawle, Dongsheng Li, Joakim E. Swedberg, Lu Wang, Dinesh C. Soares, David Harrich

**Affiliations:** a Department of Cell and Molecular Biology, QIMR Berghofer Medical Research Institute, Herston, Queensland, Australia; b School of Chemistry and Molecular Biosciences, University of Queensland, St. Lucia, Queensland, Australia; c Institute for Molecular Biosciences, University of Queensland, St. Lucia, Queensland, Australia; d MRC Institute of Genetics and Molecular Medicine, Western General Hospital, University of Edinburgh, Edinburgh, United Kingdom; Rutgers-Robert Wood Johnson Medical School; Columbia University

**Keywords:** uncoating, eEF1A, human immunodeficiency virus, reverse transcriptase, virus-host interactions

## Abstract

Once HIV-1 enters a cell, the viral core is uncoated by a poorly understood mechanism and the HIV-1 genomic RNA is reverse transcribed into DNA. Host cell factors are essential for these processes, although very few reverse transcription complex binding host cell factors have been convincingly shown to affect uncoating or reverse transcription. We previously reported that cellular eukaryotic translation elongation factor 1A (eEF1A) interacts tightly and directly with HIV-1 reverse transcriptase (RT) for more efficient reverse transcription. Here we report that the surface-exposed acidic residues in the HIV-1 RT thumb domain alpha-J helix and flanking regions are important for interaction with eEF1A. Mutation of surface-exposed acidic thumb domain residues D250, E297, E298, and E300 to arginine resulted in various levels of impairment of the interaction between RT and eEF1A. This indicates that this negatively charged region in the RT thumb domain is important for interaction with the positively charged eEF1A protein. The impairment of RT and eEF1A interaction by the RT mutations correlated with the efficiency of reverse transcription, uncoating, and infectivity. The best example of this is the strictly conserved E300 residue, where mutation significantly impaired the interaction of RT with eEF1A and virus replication in CD4^+^ T cells without affecting *in vitro* RT catalytic activity, RT heterodimerization, or RNase H activity. This study demonstrated that the interaction between surface-exposed acidic residues of the RT thumb domain and eEF1A is important for HIV-1 uncoating, reverse transcription, and replication.

## IMPORTANCE

HIV-1, like all viruses, requires host cell proteins for its replication. Understanding the mechanisms behind virus-host interactions can lay the foundation for future novel therapeutic developments. Our lab has identified eEF1A as a key HIV-1 RT binding host protein that is important for the reverse transcription of HIV-1 genomic RNA into DNA. Here we identify the first surface-exposed RT residues that underpin interactions with eEF1A. Mutation of one strictly conserved RT residue (E300R) delayed reverse transcription and viral core uncoating and strongly inhibited HIV-1 replication in CD4^+^ T cells. This study advances the structural and mechanistic detail of the key RT-eEF1A interaction in HIV-1 infection and indicates its importance in uncoating for the first time. This provides a further basis for the development of an RT-eEF1A interaction-inhibiting anti-HIV-1 drug and suggests that the surface-exposed acidic patch of the RT thumb domain may be an attractive drug target.

## INTRODUCTION

After HIV-1 fusion with the cell membrane, the viral core is released into the cytoplasm of the cell, where HIV-1 utilizes host factors for replication (1). The trafficking of the HIV-1 core and reverse transcription complex (RTC) from the cell periphery to the nucleus is tightly regulated by viral and host proteins (2), but the process is poorly understood. The RTC is comprised of several viral and cellular proteins and facilitates conversion of the HIV-1 genomic RNA into double-stranded DNA, a process known as reverse transcription (3, 4). Evidence is emerging that reverse transcription may be tightly linked with the progression of HIV-1 uncoating in the cytoplasm, where inhibition of reverse transcription delays uncoating (5, 6). While there are different proposed models of HIV-1 uncoating, the emerging evidence supports a model where the hexameric capsid (CA) lattice is disassembled, resulting in loss of integrity of the conical core within the first 1 to 4 h postinfection, depending on the cell type (5, 7); however, the CA remains associated with HIV-1 replication complexes during nuclear trafficking and after nuclear entry (8–11). Despite mechanistic disparities in the literature, it is accepted that uncoating is highly regulated to achieve optimal timing kinetics, where too rapid or too delayed uncoating is detrimental to HIV-1 replication (12). However, how uncoating kinetics is regulated is not completely understood.

It is well known that host cellular factors are crucial for reverse transcription (3), and more recent evidence suggests that cellular factors may be important for the regulation of HIV-1 uncoating (13, 14). Identifying how specific host cell factors facilitate optimal uncoating and reverse transcription is important to better understand these early stages of HIV-1 replication. This could lead to the development of a different class of drugs that target virus-host interactions required for early stages of HIV-1 replication (15, 16). Our earlier study showed that eukaryotic translation elongation factor 1A (eEF1A) is required for early stages of HIV-1 replication (17, 18).

The highly abundant cellular protein eEF1A is involved in the delivery of aminoacylated tRNAs to the elongating ribosome and has also been implicated in the replication of several different RNA viruses (reviewed in reference 19). Our previous studies demonstrated that eEF1A binds tightly (binding affinity [*K*_*D*_] = 3 to 4 nM) and directly to HIV-1 reverse transcriptase (RT) and is likely the predominant RT binding cellular protein (17, 18). Domain mapping revealed that the thumb and connection domains of HIV-1 RT are important for binding to eEF1A, and mutagenesis of the RT thumb domain showed that the W252A and L303A mutations significantly impair binding to eEF1A (18). However, protein modeling and bioinformatic predictions indicated that these amino acids cannot directly support interaction with eEF1A because they are buried within the core of the HIV-1 RT protein (18). Mutation of these residues to alanine was predicted to severely destabilize the RT protein structure (18) and therefore possibly disrupt the RT region that directly contacts eEF1A, but this region has yet to be identified.

Using small interfering RNA (siRNA) knockdown of eEF1A and an eEF1A inhibitor called didemnin B, Warren et al. and Li et al. revealed that the RT-eEF1A interaction is crucial for HIV-1 reverse transcription, particularly late-stage DNA synthesis (17, 18). While the mechanism behind the important role of eEF1A in reverse transcription has yet to be fully elucidated, siRNA knockdown of eEF1A and didemnin B treatment resulted in reduced levels of RTCs in infected cells, suggesting that eEF1A may be an RTC-stabilizing factor (17, 18). However, whether the HIV-1 RT-eEF1A interaction is important for uncoating has yet to be investigated.

This study aimed to better define how HIV-1 RT interacts with eEF1A by identifying surface-exposed RT residues that facilitate direct interaction with eEF1A and to determine whether this interaction is important for optimal HIV-1 uncoating kinetics. We found that a cluster of acidic residues on the surface of the HIV-1 RT thumb domain is important for interaction with positively charged cellular eEF1A. Arginine mutagenesis of these surface-exposed acidic amino acids resulted in impairment of the RT-eEF1A interaction, delayed HIV-1 uncoating, reduced reverse transcription efficiency, and resulted in defective virus replication in CD4^+^ T cells.

## RESULTS

### *In silico* simulations reveal that the W252A and L303A mutations disrupt a surface-exposed acidic patch of residues in the alpha-J helix and flanking regions of the HIV-1 RT thumb domain.

**FIG 1:**
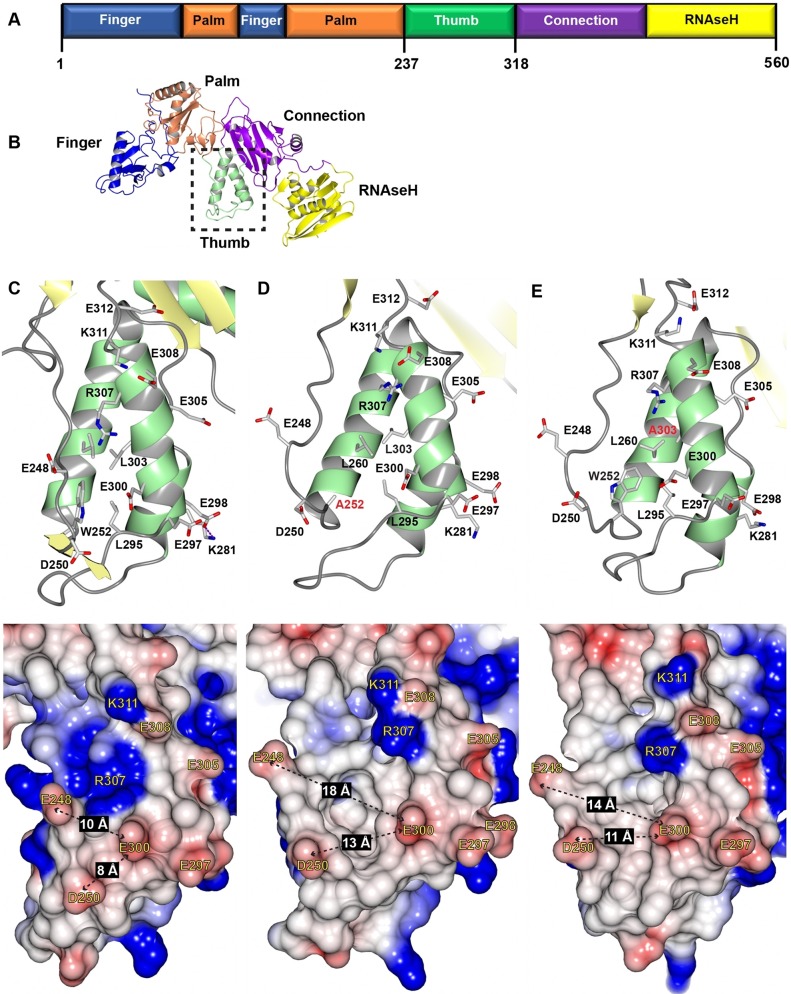
W252A and L303A mutations disrupt a surface-exposed acidic region in the alpha-J helix of the HIV-1 thumb domain. (A) Schematic representation of the HIV-1 RTp66 domains. (B) Ribbon schematic representation of HIV-1 RTp66 colored by domains. Molecular dynamic simulations of the HIV-1 RTp66 domain showing ribbon schematics and surface representation of the thumb domain of the WT (C), the W252A mutant (D), and the L303A mutant (E). Secondary-structure β-strands and α-helices (top) are yellow and green, respectively, whereas atoms are shown as stick models of carbon (gray), oxygen (red), and nitrogen (blue). Molecular surfaces (bottom) are colored by charge as follows: positive, blue; neutral, white; negative, red. Distances (in angstroms) between key residues are shown with dashed black arrows.

**FIG 2:**
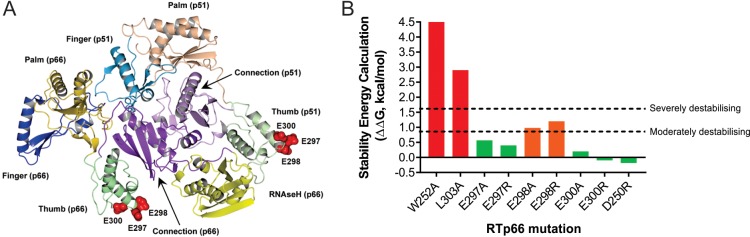
Ribbon diagram highlighting the E297, E298, and E300 residues in the HIV-1 RT thumb domain and stability energy calculations of acidic-patch mutants. (A) The side chains of the E297, E298, and E300 amino acid residues in the HIV-1 RTp51 and RTp66 alpha-J helix of the thumb domain are represented by red spheres in the RT heterodimer ribbon diagram. (B) Stability energy calculation for HIV-1 RTp66 in the context of the heterodimer with the indicated mutations in the RT thumb domain as calculated by the FoldX software. The threshold for moderate destabilization (orange) was 0.8 kcal/mol, and the threshold for severe destabilization (red) was 1.6 kcal/mol, whereas <0.8 kcal/mol was considered to have no or a minimal impact on stability (green).

Our previous study indicated that the W252A and L303A mutations reduce the HIV-1 RT interaction with cellular eEF1A, likely through changes in the structure of RT (18). Therefore, *in silico* simulations of these mutations were performed to analyze their specific effects on the structure of RT (Fig. 1A and B) to predict a surface region of RT that may interact with eEF1A. Our simulations show that the W252A and L303A mutations disrupted the peptide backbone across residues 250 to 253, which have a β-strand-like conformation, and resulted in increased flexibility in the unstructured loop spanning residues 244 to 249 (Fig. 1C compared to D and E). There were conformational changes in surface-exposed residues in the thumb domain, highlighted by an increase in the distance between the E300 residue and the E248 and D250 residues (Fig. 1C compared to D and E). Furthermore, the W252A and L303A mutations were predicted to greatly reduce the frequency of the salt bridge between E298 and K281 and also alter the hydrogen bonding pattern of several surface-exposed electrically charged residues in the thumb domain (Fig. 1; see Table S1 in the supplemental material). Overall, these data suggest that the W252A and L303A mutations result in changes in the local conformation and intramolecular interactions of the surface-exposed residues of the HIV-1 thumb domain. Since the HIV-1 RT thumb surface contains a cluster of acidic residues and eEF1A is a highly basic protein (20), these acidic residues were investigated further. Analysis of an RT multiple-sequence alignment revealed that while D250 and E297 are not conserved, E298 is largely conserved and E300 is strictly conserved among HIV-1 sequences (Fig. S1). E297, E298, and E300 are a highly surface-exposed (Fig. S1) cluster of acidic residues in the alpha-J helix of the RT thumb domain and do not contact other RT domains in the crystal structure (Fig. 2A). Therefore, to investigate if these residues are important for interaction with eEF1A, the D250, E297, E298, and E300 residues were mutated to alanine (A) and arginine (R).

10.1128/mBio.00316-18.1 FIG S1  Amino acid alignment and surface exposure of RT thumb domain residues. Amino acid position numbers correspond to the HIV-1 RT query sequence. Strictly conserved residue positions are highlighted with a red background. Largely conserved residue positions are shown on a yellow background. Secondary structure is shown above the alignment; e.g., the three α-helices that make up the thumb domain are labeled (α5, α6/n6,and α7; also called αH, αI, and αJ, respectively) from the N terminus. The solvent accessibility of each residue position is shown below the alignment (white, buried; cyan, intermediate; blue, highly exposed), which corresponds to p66 chain A of the heterodimer. The image shown was prepared and annotated with EsPript version 3 (62). Download FIG S1, PDF file, 0.04 MB. Copyright © 2018 Rawle et al. 2018 Rawle et al. This content is distributed under the terms of the Creative Commons Attribution 4.0 International license.

10.1128/mBio.00316-18.7 TABLE S1  Hydrogen bonding frequency residues in the thumb domain of WT and mutant RT subunit p66 during molecular dynamics simulations. Mutated residues are red, and the color code used for the frequency is shown at the bottom. Download TABLE S1, DOCX file, 0.02 MB. Copyright © 2018 Rawle et al. 2018 Rawle et al. This content is distributed under the terms of the Creative Commons Attribution 4.0 International license.

Calculations of changes in the thermodynamic stability of the RT heterodimer caused by RTp66 mutagenesis were performed by using the empirical force field FoldX program, which showed that the W252A and L303A mutations are severely destabilizing (Fig. 2B). Mutation of the E298 residue to alanine (A) or arginine (R) was predicted to result in moderate destabilization (Fig. 2B). Mutation of D250, E297, or E300 to alanine or arginine was predicted to have no or a minimal effect on overall RT heterodimer stability (Fig. 2B). The heterodimer stability energy calculations for RTp66 (Fig. 2B) and RTp51 (Fig. S2) mutations showed similar results. Molecular dynamic simulations supported the FoldX results in that the E298R mutation (but not the E300R mutation) disrupted the peptide backbone across residues 250 to 253, similarly to the W252A and L303A mutations, and thereby caused a substantial redistribution of the positive and negative charges on the surface of the thumb domain (Fig. S3; Table S1). It is unclear whether the β-strand-like conformation of the peptide backbone across residues 250 to 253 or increased flexibility in the unstructured loop spanning residues 244 to 249 has any specific importance in the interaction of RT with eEF1A; however, it is a clear marker of more widespread structural changes in the W252A, L303A, and E298R mutations compared to E300R in our molecular dynamic simulations.

10.1128/mBio.00316-18.2 FIG S2  Stability energy calculations for mutations in the RTp51 subunit in the context of the heterodimer. Stability energy calculation for HIV-1 RTp51 in the context of the heterodimer with the indicated mutations in the RT thumb domain as calculated by the FoldX software. The threshold for moderate destabilization (orange) was 0.8 kcal/mol, and the threshold for severe destabilization (red) was 1.6 kcal/mol, whereas <0.8 kcal/mol was considered to have no or a minimal impact on stability (green). Download FIG S2, TIF file, 0.2 MB. Copyright © 2018 Rawle et al. 2018 Rawle et al. This content is distributed under the terms of the Creative Commons Attribution 4.0 International license.

10.1128/mBio.00316-18.3 FIG S3  E298R mutation causes more structural change in the RTp66 thumb domain than the E300R mutation. Molecular dynamic simulations of the HIV-1 RTp66 domain showing ribbon schematics and surface representation of the thumb domain of the WT (A), the E298R mutant (B), and the E300R mutant (C). Secondary structure β-strands and α-helices (top) are yellow and green, respectively, whereas atoms are shown as stick models of carbon (gray), oxygen (red), and nitrogen (blue). Molecular surfaces are colored by charge as follows: positive, blue; neutral, white; negative, red. Distances (in angstroms) between key residues are shown with dashed black arrows. Download FIG S3, JPG file, 1.8 MB. Copyright © 2018 Rawle et al. 2018 Rawle et al. This content is distributed under the terms of the Creative Commons Attribution 4.0 International license.

### Mutation of surface-exposed acidic residues in the alpha-J helix and flanking regions of the RT thumb domain reduces the interaction with eEF1A.

**FIG 3:**
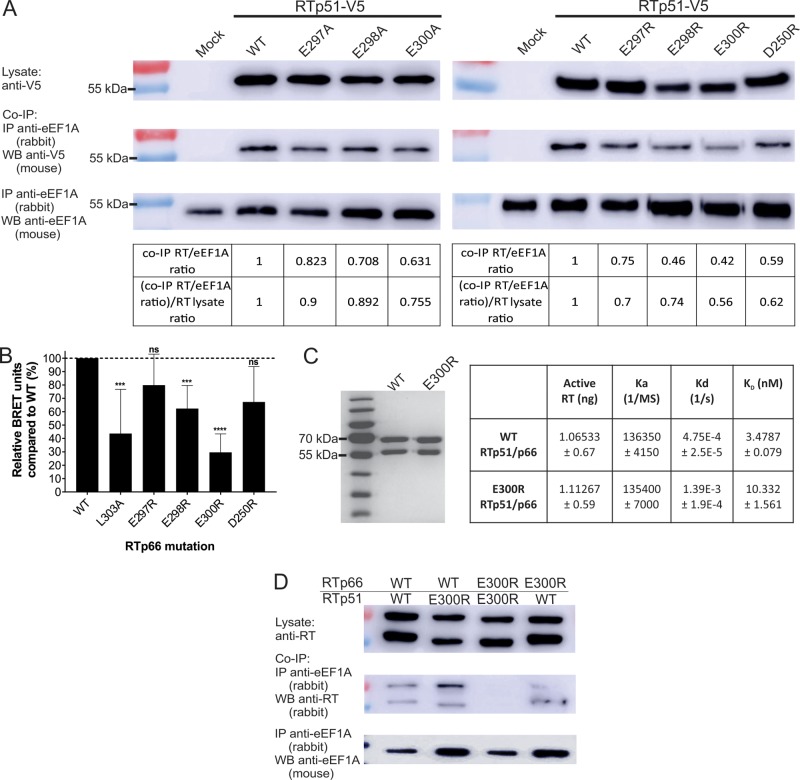
Mutations of surface-exposed acidic residues in the RT thumb domain to basic residues result in reduction of HIV-1 RT-eEF1A interaction. (A) Lysates of HEK293T cells expressing WT or mutant recombinant HIV-1 RTp51 (top) were immunoprecipitated with an anti-eEF1A antibody. HIV-1 RT protein levels in the immunoprecipitate were analyzed by Western blotting with an anti-V5 antibody (middle), and levels of eEF1A in the immunoprecipitate were analyzed by Western blotting with an anti-eEF1A antibody (bottom). ImageJ was used to calculate the ratio of the density of the co-IP RT bands to the co-IP eEF1A bands (table, top row), and this was also normalized to the density of the lysate RT bands (table, bottom row). (B) NanoBRET was performed with HEK293T cells cotransfected with WT or mutant RTp66-NanoLuc and HaloTag-eEF1A, BRET units were calculated, and the BRET units for the RSV P-NanoLuc-HaloTag-eEF1A interaction negative control were subtracted. Data are the mean number of relative BRET units compared to the WT for the RTp66 mutants (*n* = 7 for WT, E297R, E298R, and E300R; *n* = 3 for D250R and L303A), and error bars represent the standard error of the mean. ***, *P* < 0.001; ****, *P* < 0.0001. (C) WT or E300R RTp51/p66 heterodimer was purified from *E. coli* and analyzed by SDS-PAGE and Coomassie staining. RT catalytic activity (nanograms of active RT) of the purified proteins was measured by a standard RT colorimetric assay. A BLI assay on the Octet Red machine was used to determine the association (*K*_*a*_) and dissociation (*K*_*d*_) constants of purified WT and E300R mutant RT heterodimer with biotinylated eEF1A immobilized on a streptavidin-coated biosensor. The *K*_*a*_ is the rate of RT complex formation with eEF1A per second in a 1 M solution, and the *K*_*d*_ is the fraction of complexes decayed per second. The *K*_*a*_ divided by the *K*_*d*_ is the binding affinity (*K*_*D*_). Data are the mean of two independent experiments ± the average deviation. (D) Lysates of HEK293T cells expressing the indicated combinations of WT or E300R RTp51 or RTp66 (top) were immunoprecipitated with an anti-eEF1A antibody. HIV-1 RT protein levels in the immunoprecipitate were analyzed by Western blotting with an anti-RT antibody (middle), and levels of eEF1A in the immunoprecipitate were analyzed by Western blotting with an anti-eEF1A antibody (bottom).

To determine if surface-exposed acidic residues in or adjacent to the thumb domain alpha-J helix interact with eEF1A, we mutated V5 epitope-tagged RTp51 amino acids D250, E297, E298, and E300 to alanine or arginine. Each protein was expressed in HEK293T cells, and cell lysates were prepared. Western blot analysis of cell lysates showed that RT was expressed at similar levels with all of the mutations (Fig. 3A). Coimmunoprecipitation (co-IP) of cell lysates with an anti-eEF1A antibody was performed, and each mutation was analyzed by ImageJ densitometry to calculate the ratio of coimmunoprecipitated RT normalized to the immunoprecipitated eEF1A (Fig. 3A, table, top row), and this ratio was normalized to RT levels in the lysate (Fig. 3A, table, bottom row). The results showed that each individual alanine mutation of RT had a small effect on the amount of coimmunoprecipitated RT (Fig. 3A, left side), where a trend toward reduced levels of captured mutant RT co-IP was observed, especially for E300A. Mutation of the individual D250, E297, E298, or E300 residue to arginine produced a reduced level of coimmunoprecipitated RT, and the E300R mutation caused the most defective interaction with eEF1A (Fig. 3A, right side).

To confirm the co-IP results in RTp66, the NanoBRET (Promega) assay was performed with HEK293T cells cotransfected with an HIV-1 RTp66-NanoLuc expression vector and a HaloTag-eEF1A expression vector (Fig. S4A). Energy transfer from NanoLuc to the HaloTag binding ligand occurs when their RTp66 and eEF1A fusion proteins, respectively, are in very close proximity (~5 nm) as a result of protein interaction (Fig. S4B) (21). Mutant RT expression was internally controlled by measuring NanoLuc emission (450 nm), and this was divided by the fluorescence emission (610 nm) of the HaloTag binding ligand, which is activated by energy transfer from NanoLuc excitation. The bioluminescence resonance energy transfer (BRET) ratio was calculated for each RT mutation and normalized to the BRET ratio for the wild-type (WT) RT interaction with eEF1A (100%), thus calculating the mutant RT interaction as a percentage of the WT RT interaction (Fig. 3B). The cotransfection of respiratory syncytial virus (RSV) phosphoprotein (P)-NanoLuc with HaloTag-eEF1A was used as the negative control, as we have previously shown that RSV P does not interact with eEF1A by co-IP (22), and this BRET ratio was subtracted from the RT-eEF1A interaction BRET ratio for each mutant RT. We found that the E297R and D250R mutations reduced the interaction with recombinant eEF1A by approximately 20 and 33% compared to WT RT, respectively, although this was not statistically significant (Fig. 3B). However, the E298R and E300R mutations significantly reduced the interaction with recombinant eEF1A by 40 and 70% compared to WT RT, respectively (Fig. 3B). NanoBRET also confirmed that RTp66 with the L303A mutation had a reduced interaction with recombinant eEF1A (Fig. 3B), matching previously reported co-IP and biolayer interferometry (BLI) experiments (18). Thus, NanoBRET assays support and mirror the co-IP results and indicate that mutation of surface-exposed acidic residues to arginine resulted in impairment of the interaction of HIV-1 RT with eEF1A, with E300R being the most significant example of this.

10.1128/mBio.00316-18.4 FIG S4  NanoBRET schematic of RTp66 interacting with eEF1A. (A) Schematic of the RTp66-NanoLuc and HaloTag-eEF1A plasmid constructs. (B) When RTp66-NanoLuc interacts with HaloTag-eEF1A, the NanoLuc 450-nm emission excites the HaloTag binding ligand, which emits a 618-nm fluorescent signal. Download FIG S4, TIF file, 6.2 MB. Copyright © 2018 Rawle et al. 2018 Rawle et al. This content is distributed under the terms of the Creative Commons Attribution 4.0 International license.

Since the E300R RT mutation reduced binding to eEF1A the most, it was used to confirm the co-IP and NanoBRET binding results in an *in vitro* system with the RTp51/p66 heterodimer. Before this, a cell-based system called mammalian protein-protein interaction trap (MAPPIT) was used to determine if the E300R mutation affects RTp51 and RTp66 heterodimerization, which induces luciferase expression in the MAPPIT system as previously described (18). A known RT heterodimer inhibitory mutation, L234A (23, 24), substantially reduced the MAPPIT luciferase signal below that of the WT RT heterodimer interaction (Fig. S5), thus validating the system. Luciferase expression was similar between the cotransfection of both WT RT heterodimer subunits and both E300R mutant RT heterodimer subunits, indicating that RTp51 and RTp66 heterodimerization was not affected by the E300R mutation (Fig. S5). A WT and E300R mutant RT heterodimer was purified from *Escherichia coli* by previously described methods (25), and Coomassie stain analysis indicated that the purified proteins were free of contaminants (Fig. 3C). Similar concentrations of the purified WT and E300R mutant RT heterodimer contained similar amounts of active RT measured in a standard *in vitro* assay with a homopolymer poly(A) DNA template and oligo(dT) primers (Fig. 3C). These proteins were then subjected to a BLI assay where biotinylated eEF1A was immobilized on a streptavidin-coated biosensor as previously described (18). The biosensor was then incubated in 90 nM WT or E300R mutant RT heterodimer, and these proteins had similar association rate constants (Fig. 3C). However, the E300R mutant RTp51/p66 heterodimer dissociation rate constant was approximately 3-fold higher than that of the WT protein (Fig. 3C). This led to the E300R mutant RT heterodimer having 3-fold weaker eEF1A binding affinity than the WT (Fig. 3C). This is in agreement with the co-IP and NanoBRET finding that the E300R mutation impairs the interaction of RT with eEF1A.

10.1128/mBio.00316-18.5 FIG S5  MAPPIT assay showing that E300R does not affect the heterodimerization of RTp51 and RTp66. A leptin receptor deficient for STAT3 recruitment is fused C terminally to a bait protein (RTp51), and a prey protein (RTp66) is N-terminally fused to a gp130 chain with four functional STAT3 recruitment sites. In the presence of leptin, the interaction between the RTp51 bait and the RTp66 prey leads to complementation of STAT3 signaling and activation of a reporter luciferase gene expressed by the rPAP1 promoter. MAPPIT bait and prey WT RT, mutant RT, or MYD88 and SVT negative-control plasmids were cotransfected with the pXP2D2-rPAP1-luciferase reporter plasmid in the combinations indicated; leptin (100 ng/ml) was added at 24 h posttransfection; and the mixture was incubated for a further 24 h before cell lysate was used in firefly luciferase assays. Data are the mean relative luciferase activity units in two independent experiments performed in triplicate, and error bars represent the standard error of the mean. Download FIG S5, TIF file, 0.2 MB. Copyright © 2018 Rawle et al. 2018 Rawle et al. This content is distributed under the terms of the Creative Commons Attribution 4.0 International license.

We performed additional co-IP experiments to investigate the ability of RTp51 and RTp66 heterodimer subunits to interact with eEF1A. The WT and E300R mutant RT heterodimer subunits were cotransfected in all possible combinations, and an anti-eEF1A antibody was used to immunoprecipitate eEF1A in cell lysates. Western blot assays were performed with an anti-RT antibody to detect coimmunoprecipitated RT heterodimer subunits. The E300R mutant RT heterodimer subunits had substantially reduced co-IP with eEF1A compared to the WT RT heterodimer subunits (Fig. 3D). However, when WT RTp51 was cotransfected with E300R mutant RTp66 or E300R mutant RTp51 was cotransfected with WT RTp66, both RT subunits were coimmunoprecipitated with eEF1A (Fig. 3D). Hence, expression of either WT RT subunit can rescue the co-IP of the counterpart E300R mutant RT heterodimer subunit. We interpret this result as indicating that eEF1A can bind to the RT heterodimer by interaction with the thumb domain on either WT RT heterodimer subunit.

### HIV-1 assembly, packaging, maturation, *in vitro* RT catalytic activity, and ERT are unaffected by the E300R RT mutation.

**FIG 4:**
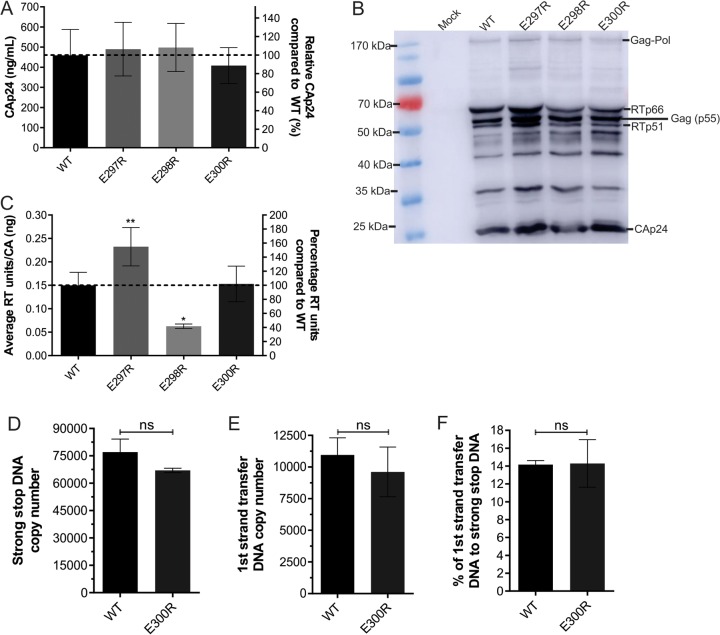
Characterization of HIV-1 harboring E297R, E298R, and E300R mutations. (A) Amounts of HIV-1 CA present in culture supernatant as determined by CA ELISA after HEK293T cotransfection with WT or mutant NL4.3 proviral plasmid. (B) Western blot analysis of concentrated WT or mutant HIV-1 particles normalized to CA, detected with an anti-HIV-1 (human serum, NIH AIDS reagent). (C) The *in vitro* RT catalytic activity of WT or mutant RT from lysed HIV-1_NL4.3_ and HIV-1_NL4_._3_-Δenv-eGFP virions, normalized to CA levels, as measured by a standard RT colorimetric assay. Data are the mean of at least four independent experiments, and error bars represent the standard error of the mean. *, *P* < 0.05; **, *P* < 0.01. ERT assays were performed with similar amounts of WT and E300R HIV-1 virions incubated with or without dNTPs for 6 h, and qPCR was used to measure ssDNA (D) and first strand transfer DNA (E). The DNA copy number of virions incubated without dNTPs was subtracted from the DNA copy number of virions incubated with dNTPs. The result was used to calculate the percentage of first strand transfer DNA to ssDNA (F). Data are the mean of two independent experiments with qPCR performed in duplicate, and error bars represent the standard error of the mean. An unpaired *t* test showed no statistical significance (ns).

The E297R, E298R, and E300R mutations, which represent weak, medium, and strong impairment of the RT-eEF1A interaction, respectively, were introduced into the HIV-1_NL4.3_ and HIV-1_NL4_._3_-Δenv-eGFP reporter proviral plasmids to produce infectious HIV-1 particles in HEK293T cells. HIV-1_NL4_._3_-Δenv-eGFP was VSV-G pseudotyped. The production of particles by each WT or mutant HIV-1 strain was measured with a CA enzyme-linked immunosorbent assay (ELISA), and no significant difference in virus production was observed (Fig. 4A). Western blot analysis of similar amounts of total CA of partially purified virions with an anti-HIV-1 polyclonal antibody showed that HIV-1 proteins for WT and mutant HIV-1 virions were present (Fig. 4B), suggesting that the RT mutations did not affect gag-pol processing and virus maturation as has been described for other RT mutations (26). Enzymatic activity of WT or mutant RT in lysed virions was subjected to a standard *in vitro* assay with a homopolymer poly(A) DNA template and oligo(dT) primers. The assay showed that the E298R mutation caused a significant 60% reduction in RT catalytic activity, the E297R mutation caused a significant 50% increase in RT activity, and the E300R mutation had no significant effect on *in vitro* RT catalytic activity compared to the WT (Fig. 4C). The virion E300R mutant RT activity compared to that of the WT correlated with results obtained with a purified recombinant RT heterodimer (Fig. 3C), showing that the overall enzymatic activity is similar. To further investigate the activity of RT in the E300R virions, an endogenous reverse transcription (ERT) assay was performed with similar amounts of WT and E300R virions incubated with deoxynucleoside triphosphates (dNTPs). After 6 h, the virions were lysed and DNA was purified and used in a quantitative PCR (qPCR) to measure strong-stop DNA (ssDNA) and first strand transfer DNA. This showed that there was no statistically significant difference between the WT and E300R mutant HIV-1 strains in the level of ssDNA (Fig. 4D), the level of first strand transfer DNA (Fig. 4E), or the percentage of ssDNA that is first strand transfer DNA (Fig. 4F) (*P* > 0.05). This further indicates that the E300R mutation did not affect RT enzymatic activity, including RNase H activity. As all three mutations have variable effects on the RT-eEF1A interaction and *in vitro* RT catalytic activity, their effect on HIV-1 replication was investigated.

### HIV-1 with the E298R and E300R RT mutations has impaired infectivity, delayed reverse transcription, and reduced reverse transcription efficiency in HeLa cells.

**FIG 5:**
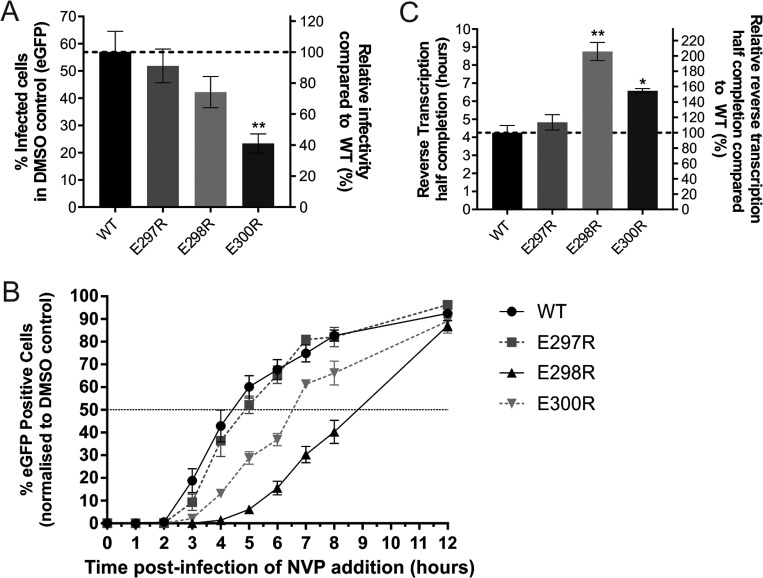
RT thumb domain mutations delay reverse transcription completion as measured by nevirapine addition assay. (A) Percentage of HeLa cells expressing eGFP at 48 h postinfection with 5 ng of CA of WT or RT-mutated HIV-1. (B) Nevirapine addition assay performed with HeLa cells. HeLa cells were infected with equal CA amounts of HIV-1 in a 96-well plate, and 5 µM nevirapine was added at the postinfection times indicated. The percentage of successfully infected cells was measured by eGFP detection by flow cytometry at 2 days postinfection. The percentage of eGFP at each time point was normalized to the DMSO-only control, which represents 100% completion of reverse transcription. (C) The time at which 50% of the cells were infected with WT or mutant HIV-1 that had completed reverse transcription as determined by nevirapine addition assay. The data are the mean of four independent experiments, and error bars represent the standard error of the mean. *, *P* < 0.05; **, *P* < 0.01.

The infectivity of VSV-G-pseudotyped HIV-1_NL4_._3_-Δenv-eGFP containing each RT mutation was examined by infecting HeLa cells with the same amount of HIV-1 normalized to the total CA, and the percentage of enhanced green fluorescent protein (eGFP)-positive cells at 48 h postinfection was measured by flow cytometry. WT HIV-1 infection produced 57% eGFP-positive cells, while E297R, E298R, and E300R mutant infections produced 52, 42, and 23% eGFP-positive cells, respectively (mean of six experiments) (Fig. 5A). An interpretation of the reduced infectivity of E298R is complicated, as this virus had less *in vitro* RT catalytic activity than the others (Fig. 4C). However, the E300R mutation had significantly (*P* < 0.05) reduced HIV-1 infectivity, even though RT *in vitro* catalytic activity and ERT were unaffected. In comparison, E297R mutant HIV-1_NL4_._3_-Δenv-eGFP had increased RT catalytic activity *in vitro*; however, its infectivity was not significantly different from that of the WT virus. These results suggest that, after taking into account the RT enzymatic activity changes, only the E300R mutation, which had the greatest negative effect on the RT-eEF1A interaction, also significantly affects virus infectivity in HeLa cells.

Complementary assays were employed to determine if the HIV-1 RT acidic mutations had temporal effects on reverse transcription completion in HeLa cells. A nevirapine addition assay was used to track the relative percentage of infected cells that had completed reverse transcription at specific time points postinfection with the WT and RT mutant viruses. The addition of nevirapine stops reverse transcription and inhibits successful infection by HIV-1 if reverse transcription is incomplete. Conversely, HIV-1 with completed reverse transcription will not be affected by nevirapine and integrate into the cellular DNA, which can then express eGFP. This experiment measured the percentage of eGFP^+^ cells at 48 h postinfection with each virus in the presence of nevirapine added at the time points shown (Fig. 5B), normalized to the dimethyl sulfoxide (DMSO) control (set to 100%) (Fig. 5A). The results show that the E298R mutant, which had the lowest level of RT catalytic activity *in vitro* (Fig. 4C), had the most delayed completion of reverse transcription, with 20 to 50% less completion of reverse transcription between 3 and 8 h postinfection than the WT virus (Fig. 5B). The reverse transcription kinetics of the E297R mutant were similar to those of the WT virus (Fig. 5B). HIV-1 with the RT E300R mutation had 10 to 30% less reverse transcription completion between 3 and 6 h postinfection than the WT (Fig. 5B). The time when half of the viral RTCs had completed reverse transcription was approximately 2 h later for HIV-1 with the E300R mutation (50% delay), 4 h later for HIV-1 with the E298R mutation (100% delay), and 30 min later for E297R (14% delay) than for WT HIV-1 (Fig. 5C).

To determine which stage of reverse transcription is affected by the mutations, HeLa cells were infected with the same amount of CA of WT or mutant HIV-1 for 4 h and the cells were lysed. The levels of HIV-1 ssDNA, first strand transfer DNA, and late-stage DNA were measured by qPCR. HIV-1 containing each of the E297R, E298R, and E300R mutations had fewer copies of ssDNA, first strand transfer DNA, and late-stage DNA at 4 h postinfection than WT HIV-1 (Table 1). While HIV-1 with WT, E297R mutant, and E300R mutant RT had at least 90% completion of first strand transfer, HIV-1 with the E298R mutation in RT had reduced first strand transfer efficiency (Table 1), confirming enzymatic defects. The ratio of late-stage DNA copies to first strand transfer DNA copies was approximately 20% lower for the E298R and E300R mutants than for the WT and the E297R mutant, indicating that these viruses had reduced efficiency of late-stage DNA synthesis (Table 1). The same experiments performed with HIV-1 inactivated by heating produced <10 copies of DNA, indicating that DNA contamination was negligible. The combined evidence indicates that the E298R and E300R mutations, which reduce the RT-eEF1A interaction, result in reduced early- and late-stage reverse transcription but have a greater effect on late-stage DNA synthesis.

**TABLE 1:** Analysis of reverse transcription by WT or mutant VSV-G-pseudotyped HIV-1_NL4.3_ in HeLa cells at 4 h postinfection^a^

**Parameter**	**WT^b^**	**E297R** **mutant**^c^	**E298R** **mutant**^d^	**E300R mutant^e^**
ssDNA copy no.	184,400	151,150	25,475	74,175
1st strand DNA copy no.	172,900	150,500	20,305	77,750
1st strand/ssDNA %	93.76	99.57	79.71	104.82
Late DNA copy no.	30,260	24,835	2,883	11,165
Late/1st strand DNA copy % (% of WT value)	20.49 ± 2.99 (100 ± 0)	19.37 ± 2.87 (94.53 ± 0.25)	15.89 ± 1.69 (77.55 ± 3.59)	15.7 ± 1.55 (76.62 ± 5.44)

a Infection experiments were repeated twice with similar results, and qPCR was performed twice in duplicate. Each DNA copy number presented is from one infection experiment, and the late/1st strand DNA copy percentage and the percentage of the WT value are the mean values of both infection experiments ± the standard error of the mean. Heat-inactivated virus samples contained <10 copies.

b HIV-1_NL4_._3_.

c HIV-1_NL4_._3_-RT-E297R.

d HIV-1_NL4_._3_-RT-E298R.

e HIV-1_NL4_._3_-RT-E300R.

### HIV-1 with the E298R or E300R RT mutation has delayed uncoating kinetics in cells.

**FIG 6:**
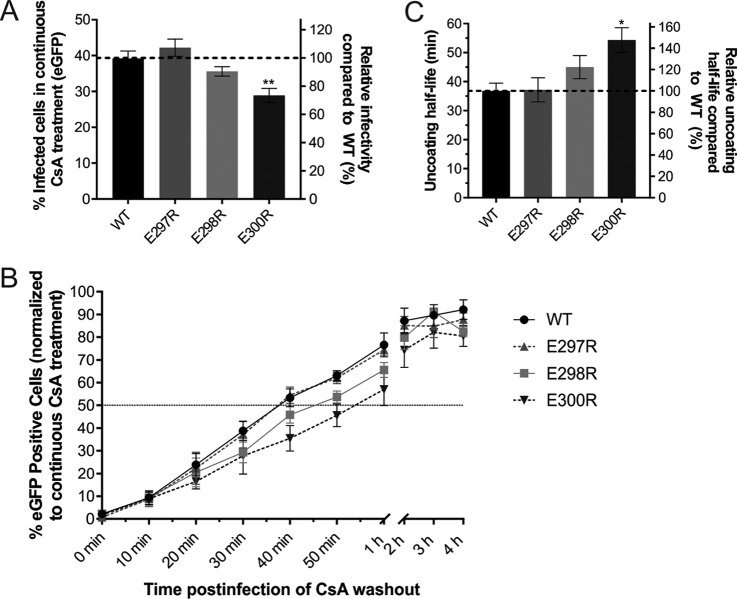
Mutation of acidic residues in the RT thumb domain delays HIV-1 uncoating kinetics as measured with the CsA washout assay. (A) Percentage of HeLa-TC cells expressing eGFP 48 h after infection with 10 ng of CA of WT or RT-mutated HIV-1 in the continuous presence of CsA. (B) CsA washout assay with HeLa-TC cells and infected with 10 ng of CA of HIV-1_NL4_._3_-Δenv-eGFP with WT or mutant RT. CsA was washed out at various times postinfection, and the percentage of successfully infected cells was measured by eGFP detection by flow cytometry 2 days postinfection. The percentage of eGFP at each time point was normalized to the continuous CsA treatment control, which represents 100% uncoated cores. (C) Uncoating half-life (minutes) determined by CsA washout assay. The data are the mean of three independent experiments performed in duplicate, and error bars represent the standard error of the mean. *, *P* < 0.05; **, *P* < 0.01.

To determine if the mutation of surface-exposed acidic residues to basic residues in the HIV-1 RT thumb domain affects optimal uncoating kinetics in cells, the cyclosporine A (CsA) washout assay was performed as previously described (11, 14, 27). The CsA washout assay utilizes HeLa cells sorted for high expression of owl monkey TRIMCypA, where the cyclophilin A (CypA) domain binds to intact hexameric CA and disassembles intact conical HIV-1 cores to restrict infection (28). CsA binds to and inhibits TRIMCypA, thus allowing intact cores to uncoat normally and infect the cell. Therefore, when CsA is removed from cells during infection, coated cores will be degraded and uncoated cores will proceed with infection, where an eGFP reporter expressed from the *env* open reading frame was used to determine the percentage of cores uncoated at specific times postinfection by flow cytometry. The HIV-1 mutants had similar infectivity in HeLa-TRIMCypA cells continuously treated with CsA (Fig. 6A) and in normal HeLa cells (Fig. 5A), where infectivity was significantly lower for E300R mutant HIV-1. To control for differences in infectivity, the eGFP percentage at each time point was normalized to that of the continuous-CsA control (set to 100%) for each HIV-1 mutant; therefore, any differences in the percentage of eGFP or the uncoating half-life between HIV-1 mutants are specific to conical core uncoating kinetics for each mutant HIV-1.

Between 60 and 80% of the cores had completed uncoating by 1 h postinfection for WT HIV-1 and all of the HIV-1 mutants tested (E297R, E298R, and E300R) (Fig. 6B). HIV-1 containing the E298R and E300R mutations had delayed uncoating kinetics compared to those of WT and E297R mutant HIV-1, with 10 and 20% fewer cells with uncoated cores between 30 and 60 min postinfection, respectively (Fig. 6B). The average uncoating half-life of the E298R mutant (45 min) and the E300R mutant (54 min) was significantly longer than that of the WT and the E297R mutant (37 min) (Fig. 6C). HIV-1 with the E300R mutation had more delayed uncoating kinetics (50%) than the E298R mutant (20%), despite the reduced *in vitro* RT catalytic activity and reverse transcription completion kinetics of E298R mutant HIV-1. The results suggest that the change in RT binding to eEF1A accounts for the delay in uncoating more than the RT catalytic activity and reverse transcription completion.

### HIV-1 with the E298R or E300R RT mutation has reduced replication in Jurkat cells, CD4^+^ T cells, and TZM-bl cells.

**FIG 7:**
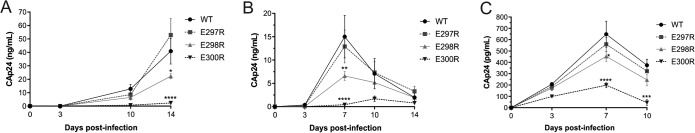
RT thumb domain mutations lead to defective HIV-1 replication in Jurkat cells, primary activated human CD4^+^ T cells, and TZM-bl cells. (A) Jurkat cells were infected with equivalent amounts of CA (50 ng of CA/10^6^ cells), and the CA in cell culture supernatant collected at 0, 3, 10, and 14 days postinfection was measured by ELISA. (B) Activated human CD4^+^ T cells were infected with equivalent amounts of CA (2 ng of CA/10^6^ cells), and the CA in cell culture supernatant collected at 0, 3, 7, 10, and 14 days postinfection was measured by ELISA. (C) HeLa cells expressing CD4 and CXCR5 (TZM-bl cells) were infected with equivalent amounts of CA, and the CA in cell culture supernatant collected at 0, 3, 7, and 10 days postinfection was measured by ELISA. The mean values are from an experiment performed in duplicate, and error bars represent the standard error of the mean. The experiment was repeated twice with similar results. *, *P* < 0.05; **, *P* < 0.01; ***, *P* < 0.001; ****, *P* < 0.0001.

Human Jurkat and primary CD4^+^ T cells were infected with WT and mutant HIV-1, and replication was measured by CA ELISA of lysed virions in cell culture supernatant at specific days postinfection. The results showed that HIV-1 with the E297R RT mutation replicated similarly to WT HIV-1 in human Jurkat (Fig. 7A) and primary CD4^+^ (Fig. 7B) T cells, whereas E298R and E300R mutant HIV-1 had significantly less replication. HIV-1 with the E298R mutation had approximately half the amount of HIV-1 produced and released into cell culture supernatant at the time point of peak HIV-1 viremia. The E300R mutant, which has RT catalytic activity similar to that of WT HIV-1 *in vitro*, produced far less HIV-1 in Jurkat cells (Fig. 7A) and activated human CD4^+^ T cells (Fig. 7B) than the WT and E297R or E298R mutant viruses. This pattern was similar in TZM-bl cells, where the E298R and E300R mutant viruses replicated significantly less than the WT virus (*P* < 0.05) and E300R mutant HIV-1 had the least replication (Fig. 7C). The combined results suggest that the E298R and E300R mutations of RT reduced the interaction with eEF1A and impaired uncoating and reverse transcription and caused defective virus replication in Jurkat cells, primary CD4^+^ T cells, and TZM-bl cells.

### Correlation of the RT-eEF1A interaction, reverse transcription, and uncoating of HIV-1 mutants.

**FIG 8:**
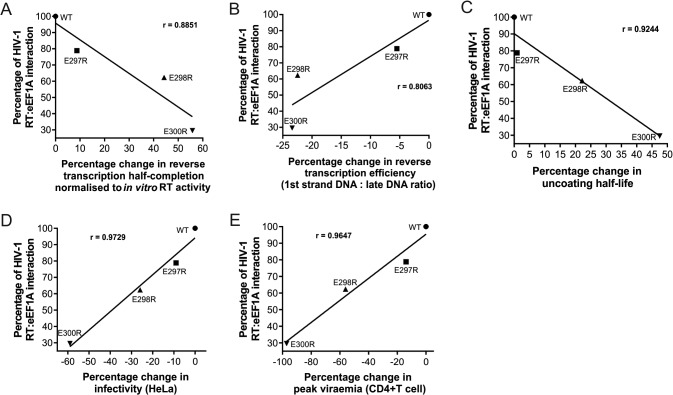
Impairment of RT-eEF1A interaction by RT thumb domain mutation correlates with defective reverse transcription completion and efficiency, delayed uncoating, and defective replication. Shown are scatterplots of WT and mutant HIV-1 RT-eEF1A interaction levels against the percent change in reverse transcription half-completion normalized to *in vitro* RT activity (A), RT-eEF1A interaction levels against the percent change in reverse transcription efficiency (B), RT-eEF1A interaction levels against the percent change in uncoating half-life (C), RT-eEF1A interaction levels against the percent change in infectivity in HeLa cells (D), and RT-eEF1A interaction levels against the percent change in peak viremia during replication in activated human CD4^+^ T cells (E).

HIV-1 reverse transcription relies on RT catalytic activity, and the *in vitro* RT catalytic activity correlated weakly with reverse transcription completion (Fig. S6A). However, the variable *in vitro* RT catalytic activity of the RT acidic residue mutations did not translate into a correlation with reverse transcription efficiency (Fig. S6B), uncoating (Fig. S6C), infectivity (Fig. S6D), or peak viremia in CD4^+^ T cells (Fig. S6E). Therefore, we performed a direct correlation analysis of the interaction of RT and eEF1A, reverse transcription, half-life of uncoating, infectivity in HeLa cells, and peak viremia in CD4^+^ T cells of the RT mutants. Since the RT catalytic activity regulates reverse transcription, the percent change in reverse transcription half-completion was normalized to *in vitro* RT catalytic activity (Fig. 4C), thus making the change in RT-eEF1A interaction the only decisive factor and allowing us to make a direct correlation. This was calculated for each acidic mutation by using the change in the relative reverse transcription half-completion percentage compared to the WT (Fig. 5C) multiplied by the fold change in *in vitro* RT catalytic activity compared to the WT (Fig. 4C). This revealed that there was a very strong correlation between the RT-eEF1A interaction levels and reverse transcription completion normalized to *in vitro* RT catalytic activity (Fig. 8A), reverse transcription efficiency (Fig. 8B), uncoating half-life (Fig. 8C), infectivity in HeLa cells (Fig. 8D), and peak viremia during replication in CD4^+^ T cells (Fig. 8E). The strong correlations suggest that the delay in reverse transcription and uncoating completion, as well as reduced infectivity and replication, caused by mutation of the acidic residues is directly linked to its impact on the interaction of RT with eEF1A.

10.1128/mBio.00316-18.6 FIG S6  Correlations for *in vitro* RT catalytic activity with HIV-1 RT mutant replication properties. Shown are scatterplots of WT or mutant HIV-1 *in vitro* RT catalytic activity against the percent change in reverse transcription half-completion (A), the percent change in reverse transcription efficiency (B), the percent change in uncoating half-life (C), the percent change in infectivity in HeLa cells (D), and the percent change in peak viremia in CD4^+^ T cells (E). Download FIG S6, TIF file, 10 MB. Copyright © 2018 Rawle et al. 2018 Rawle et al. This content is distributed under the terms of the Creative Commons Attribution 4.0 International license.

## DISCUSSION

Previously, we demonstrated that eEF1A binds tightly and directly to HIV-1 RT and that this is important for reverse transcription efficiency (18). Here, we identify the first surface-exposed residues of RT important for interaction with eEF1A and indicate the importance of this interaction for HIV-1 uncoating, reverse transcription, and replication. Complementary assays show that mutation of the surface-exposed acidic D250, E297, E298, and E300 residues in the HIV-1 RT thumb domain reduces the interaction with eEF1A at various levels. This agrees with previous predictions that there are likely multiple contact points between HIV-1 RT and cellular eEF1A that facilitate the strong interaction (18). The E300R mutation, which had the most defective binding compared to the other mutations in co-IP and NanoBRET assays, had a similar *in vitro* rate of association with eEF1A in the BLI assay. This may be because the molar ratio of RT to eEF1A is substantially higher in this *in vitro* system than a transfected or infected cell, which contains 40 to 60 µM eEF1A (29) and very small amounts of HIV-1 RT. However, the E300R mutant RT heterodimer had a greater rate of dissociation from eEF1A than the WT, suggesting that the E300R mutant RT heterodimer complexed with eEF1A is less stable. The binding affinity of the purified WT RT heterodimer for eEF1A was similar to that in a previous report (18), and the weaker eEF1A binding affinity of the E300R mutant RT heterodimer supports the co-IP and NanoBRET data. Previously, we showed that eEF1A can interact with the RTp51 subunit, the RTp66 subunit, and the heterodimer individually (18), and here we used cotransfected WT or E300R mutant RTp51 and RTp66 in a co-IP experiment that indicated that eEF1A can bind WT RTp51 or RTp66 in a heterodimer. It is unclear whether eEF1A can bind both RTp51 and RTp66 in the same heterodimer; however, the positioning of the RTp51 and RTp66 thumb domain acidic residues in the heterodimer structure does not exclude this possibility.

Our co-IP data show that mutation of these acidic residues to alanine did not significantly affect RT binding to eEF1A, whereas mutation to arginine reduced binding. Previous studies showed that a reversal-of-charge mutation is required to disrupt some strong salt bridge interactions by introducing a repulsion of two like charges (30, 31). Therefore, it is possible that introducing a positively charged arginine in the place of any of these acidic residues in HIV-1 RT may introduce various degrees of electrostatic repulsion of eEF1A, which is a highly positively charged protein with an isoelectric point of 9.1 (20). Furthermore, hydrogen bonding frequency analysis of the RTp66 mutants showed that the R298 side chain (E298R mutant) forms a salt bridge with the E297 side chain and that the R300 side chain (E300R mutant) has an increased frequency of salt bridge interactions with the E248 and D250 side chains. Therefore, these side chains in the E298R and E300R mutants would likely be less available for hydrogen bonding with eEF1A if these residues in WT RT do interact with eEF1A through salt bridge interactions. Cellular eEF1A utilizes electrostatic interactions with actin for actin bundling (32), highlighting the possibility that basic regions of eEF1A may also bind the surface-exposed acidic region of the RT thumb domain through a similar mode of interaction. However, it is possible that arginine mutagenesis may affect the interaction of RT with eEF1A by a mechanism that does not involve changes in electrostatic interactions. Further investigation is required to confirm a salt bridge interaction, including examination of the abundant surface-exposed basic patches on eEF1A (33) for RT binding.

The E297R, E298R, and E300R mutations, representing various levels of RT-eEF1A interaction impairment and predicted effect on structural stability, were introduced into HIV-1 proviral DNA for further investigation. The E297R, E298R, and E300R mutations showed weak, moderate, and strong reductions in the RT-eEF1A interaction, respectively, while causing mild, moderate, and no impairment of the predicted RT structural stability, respectively. Unexpectedly, the mild increase in predicted structural instability of the E297R mutation in RT did not reduce RT catalytic activity and instead significantly increased RT catalytic activity by 50% *in vitro*. The predicted moderate instability of RT harboring an E298R mutation resulted in a significant 60% reduction in RT catalytic activity *in vitro*, highlighting the importance of the E298 amino acid residue in RT enzyme structure and function. The reduced efficiency of first strand transfer in HIV-1 with the E298R mutation indicates a partially defective RT enzyme compared to WT, E297R mutant, and E300R mutant HIV-1, which had competent first strand transfer, supporting the *in vitro* RT catalytic activity assay results. While mutation of the E298 residue to arginine also partially reduced its interaction with eEF1A, and reverse transcription, uncoating, infectivity, and replication were also delayed and inhibited, it is hard to determine whether the cause was the reduction in *in vitro* RT catalytic activity or the reduction in the RT-eEF1A interaction. Molecular dynamic simulations revealed that the W252A, L303A, and E298R mutations caused similar widespread structural changes and also caused a major redistribution across the surface of the thumb domain, including the acidic patch of residues. This raises the possibility that the larger structure-destabilizing effect of E298 may reduce the RT-eEF1A interaction by a mechanism similar to that of the W252A and L303A mutations described previously (18), and this mutation thereby acts via a mechanism different from that of the E300R mutation.

The strict sequence conservation is another characteristic of the E300 RT residue that makes it an attractive and reliable binding site for cellular eEF1A among diverse HIV-1 isolates. Studies show that sequence conservation is greater and more common at the interface of protein-protein interactions (34, 35). The alpha-J helix in the HIV-1 thumb domain is not part of the polymerase active site or the floor of the DNA binding cleft, where only the alpha-H and alpha-I helices of the thumb domain are reported to help position the nucleic acid (36). Reverse transcription first strand transfer can only occur when HIV-1 RNase H can degrade the RNA complement to the ssDNA, indicating that RNase H activity was not affected by the E300R mutation since first strand transfer efficiency was similar to that of WT HIV-1. We were unable to find any reported functions of the strictly conserved E300 residue in reverse transcription, and our results suggest that E300 was not important for overall RT structural stability, *in vitro* enzymatic function, RNase H activity, or heterodimerization. This is in contrast to the E298R mutation, which is predicted to affect overall RT structural stability and inhibits *in vitro* enzymatic activity and RNase H activity. Therefore, it is unlikely that the uncoating and replication phenotypes of E300R mutant HIV-1 are from changes in the functionality of RT, since the known substantial effect of the E298R mutation on RT functionality affects these processes less than the E300R mutation. Purified WT and E300R mutant RT heterodimer had the same *in vitro* RT catalytic activity, but E300R mutant RT had lower eEF1A binding affinity. Therefore, we speculate that the E300 residue may be strictly conserved for the function of facilitating the interaction with cellular eEF1A, which is crucial for HIV-1 reverse transcription (17, 18), optimal uncoating, and replication. Therefore, the significantly reduced infectivity and replication of HIV-1 harboring the E300R mutation can be attributed to the defective and delayed reverse transcription and uncoating as a result of reduced interaction with eEF1A. Although the pattern is the same between cell types, the greater impact of the E300R mutation on virus replication in Jurkat and CD4^+^ T cells than in TZM-bl cells may result from a cell type difference, as WT virus replication was weak compared to that in Jurkat and CD4^+^ T cells. The E300R mutation is the only currently identified mutation of a strictly conserved and surface-exposed HIV-1 RT residue that reduces interaction with eEF1A without affecting RT protein stability or *in vitro* RT catalytic activity and provides a useful tool for investigating the role of the RT-eEF1A interaction in HIV-1 replication.

The reduced efficiency of reverse transcription in E298R and E300R mutant HIV-1, measured by the percentage of late DNA copies compared to first strand transfer DNA copies, matches the known characteristic of HIV-1 with reduced RT interaction with eEF1A during infection (18). Furthermore, the reverse transcription efficiencies of the acidic mutant viruses were not correlated with *in vitro* RT catalytic activity, suggesting that the cellular environment plays a more important role in reverse transcription efficiency (37, 38). Correlation analysis revealed that impairment of the RT-eEF1A interaction of each mutant is strongly correlated with delayed reverse transcription half-completion normalized to *in vitro* RT catalytic activity and reduced reverse transcription efficiency. The clearest example of this is the E300R mutant RT, which has no change in *in vitro* catalytic activity but a significant reduction in the interaction with eEF1A and significantly impaired HIV-1 reverse transcription kinetics and efficiency. This is consistent with our published reports that indicate that eEF1A interaction with HIV-1 RT contributes to stimulation of reverse transcription elongation, likely by stabilization of RTCs during infection (18).

A proposed and experimentally supported model of uncoating involves three stages, i.e., immediate loss of some CA and partial core opening to allow entry of cell factors required for reverse transcription, followed by gradual uncoating and loss of intact CA lattice integrity during cytoskeleton-dependent trafficking to the nucleus (simultaneous with reverse transcription) and loss of the remaining CA at the nuclear pore or in the nucleus (39–42). The CsA washout assay was used to determine when the CA lattice coating the HIV-1 core loses its integrity and functionality, thus measuring functional uncoating kinetics in cells (14, 39, 43). It measures uncoating only for cores that are infective, rather than measuring CA in the majority of cores or RTCs which are noninfective (14, 39, 43). The TRIMCypA stably expressed in HeLa cells can bind to an intact HIV-1 core with as little as 25% intact hexameric CA, indicating that the core has to be almost entirely uncoated before it is resistant to TRIMCypA restriction (43). In our experiments, WT HIV-1 had an uncoating half-life of approximately 37 min, in close agreement with another study using HeLa-TRIMCypA cells (14), indicating that the assay is reliable and reproducible. The CsA washout assay showed that the E298R and E300R HIV-1 mutants both had delayed uncoating kinetics compared to those of WT and E297R mutant HIV-1. However, HIV-1 with the E298R mutation in RT uncoated faster than the E300R RT mutant virus, despite having more substantial impairment of *in vitro* RT activity and reverse transcription completion in cells. Previous studies showed that reverse transcription completion does not solely account for changes in uncoating kinetics, since most reverse transcription does not occur until functional uncoating is complete and the RTC is formed (5, 14). However, several studies have shown that reverse transcription stages before completion have been linked to uncoating (5, 6, 12, 14, 41, 42, 44). Our data do not disagree with this but suggest that the larger negative impact of E300R mutant RT on uncoating kinetics was a result of more impaired interaction with eEF1A. This was supported by the strong correlation between NanoBRET RT-eEF1A interaction levels and uncoating half-life. It has been previously shown that the cellular milieu can affect uncoating kinetics (13, 14, 42). This is supported by evidence that the core undergoes enlargement soon after entering the cytoplasm, suggesting rapid recruitment of host cell proteins early in infection (45, 46). It is possible that soon after cell entry, eEF1A enters a partially uncoated core and binds to HIV-1 RT, and this would likely contribute to core enlargement, which may facilitate uncoating. Since eEF1A is in the HIV-1 RTC (17, 18), it is likely to be involved in RTC formation, which may also affect uncoating kinetics. Optimal uncoating kinetics within 1 h of infection are tightly linked with HIV-1 infectivity (7), which may be why E300R mutant HIV-1 is less infective than E298R mutant HIV-1.

In summary, we have found that a surface-exposed acidic patch of residues in the HIV-1 RT thumb domain, including the strictly conserved E300 residue, is important for the RT-eEF1A interaction. Mutation of these residues resulted in reduced RT interaction with eEF1A and led to a delay in reverse transcription and uncoating kinetics, as well as defective replication in human CD4^+^ T cells. This is the first time an RT binding host protein has been shown to be important for HIV-1 uncoating and highlights the importance of regulation of reverse transcription and uncoating by host members of the RTC that bind RT. Understanding the mechanism of cellular factors that are important for early stages of HIV-1 replication, such as eEF1A, could open new avenues of HIV-1 therapeutic strategies. Identification of a drug that targets the surface-exposed acidic patch of residues could block the RT-eEF1A interaction and create a new class of allosteric HIV-1 therapeutics.

## MATERIALS AND METHODS

### Molecular dynamic simulations.

Modeling of WT and mutant HIV-1 RTs was based on a crystal structure of the p66/p51 heterodimers (PDB code 4G1Q) with the p51 unit removed to reduce the system size. The WT and mutant p66 subunits were solvated with TIP3P water and neutralized with Na^+^/Cl^−^ counterions in VMD 1.9.2 (47), producing systems of approximately 65,000 atoms, including 56,000 water molecules. In the first stage, all protein heavy atoms were harmonically restrained with a force constant of 2 kcal/(mol Å^2^) before a conjugate gradient minimization of 10,000 steps was applied with NAMD 2.11 (48) and the CHARMM27 force field parameters. This was followed by heating to 298 K before simulating 2 ns (integration time step of 1 fs) under constant amount of substance (N), pressure (P), and temperature (T) conditions with periodic boundary conditions while constraining all protein heavy atoms. A Langevin thermostat with a damping coefficient of 0.5 ps^−1^ was used to maintain the system temperature. The system pressure was maintained at 1 atm with a Langevin piston barostat. The particle mesh Ewald algorithm was used to compute long-range electrostatic interactions at every time step, and nonbonded interactions were truncated smoothly between 7.5 and 9 Å. During the second stage (3 ns), the restraints on the α-carbons were retained only while covalent hydrogen bonds were constrained by the SHAKE algorithm (or the SETTLE algorithm for water), permitting an integration time step of 2 fs. For the final equilibration step (5 ns) and production simulations, only α-carbons of residues involved in hydrogen bonds between the p66/p51 heterodimers in the crystal structure (residues 85, 88, 96, 101 381, 384, 402, 407, 408, 410, 439, and 540) were constrained. The purpose of these constraints was to stabilize the p66 unit in the absence of the p51 unit.

Production runs of 400 ns were performed with ACEMD (49) under constant amount of substance (N), volume (V), and temperature (T) conditions and a Langevin thermostat with a damping coefficient of 0.1 ps^−1^ and hydrogen mass repartitioning, allowing for an integration time step of 4 fs. All other force field and simulation parameters were as described above. This process was performed three times to produce three independent production trajectories. Coordinates were saved every 2,000 simulation steps, producing 50,000 frames per trajectory. The simulation frames from 100 to 400 ns were clustered on the basis of α-carbon root mean square deviations with UCSF Chimera 1.11.1 (50), and the frames that most closely aligned with the largest cluster were selected as representative models for Fig. 1 and S2. The hydrogen bond frequency was determined with the hydrogen bond plugin in VMD with hydrogen bond lengths and angles set to 3.3 Å and 40°, respectively.

### Structure-based stability analysis of the HIV-1 RTp66 mutations.

The free-energy difference that represents the stability change in the RT p51/p66 heterodimer (PDB code 4G1Q) (51) after mutagenesis of RTp51 or RTp66 individually from the WT was determined with the empirical force field FoldX by using the YASARA molecular visualization program as previously reported (18). FoldX-predicted energy changes, i.e., ΔΔ*G* values, of >1.6 kcal/mol are considered highly significant (99% confidence interval), as they correspond to twice the standard deviation of the error in FoldX and correspond to mutations that confer severely reduced structural stability, while smaller energy changes of >0.8 kcal/mol are still considered significant (1 standard deviation; 95% confidence interval) (52–55).

### Multiple-sequence alignment.

Multiple-sequence alignment was generated with ClustalX (56). The thumb and connection domain of HIV-1 RT was used as the query sequence for a BLAST search of the SwissProt database. Related sequences with up to ~50% identity were selected, and identical redundant sequences were removed. Those sequences that had missing fragments/deletions and potential sequencing errors were also removed.

### Cell lines and virus culture.

HEK293T (ATCC) and TZM-bl (57, 58) cells were maintained in Dulbecco’s modified Eagle medium (Gibco) supplemented with 10% heat-inactivated fetal calf serum (FCS), penicillin (100 IU/ml), and streptomycin (100 µg/ml). Jurkat (clone E6-1; ATCC) and HeLa (clone CCL-2; ATCC) cells were maintained in RPMI 1640 supplemented with 10% heat-inactivated FCS, penicillin (100 IU/ml), and streptomycin (100 µg/ml). All cells were incubated at 37°C in 5% CO_2_. Stocks of HIV-1_NL4.3_ (59) with WT or mutant RT were generated by transfection of the proviral plasmid into HEK293T cells with or without a VSV-G expression vector by using X-tremeGENE HP transfection reagent (Roche). Cell culture supernatants were collected at 48 h posttransfection, filtered through a 0.45-µm filter, and stored at −80°C. HIV-1 CA levels in virus stocks and other viral samples were measured with a RETROtek HIV-1 CA antigen ELISA (ZeptoMetrix, United States) in accordance with the manufacturer’s instructions. The *in vitro* RT enzymatic activity in virus stocks was determined with a colorimetric RT assay (Roche) with a poly(A)-oligo(dT)15 template in accordance with the manufacturer’s instructions.

### Plasmids.

For expression of HIV-1 RTp51 in cells for co-IP, a destination mammalian expression vector containing a codon-optimized RT sequence with a C-terminal V5 and 6×His tag was used as previously described (18). For NanoBRET (Promega), the pFC32K-RTp66-NanoLuc expression vector was used to express RTp66 with a NanoLuc fusion at the C terminus, and a pFN21-HaloTag-eEF1A vector was used to express eEF1A with a HaloTag fusion at the N terminus. The pSicor-mCherry-T2A-Pro-TRIMCypA vector used in the CsA washout assay was constructed with a modified pSicor-mCherry-T2A backbone (Addgene no. 31845), and proline-TRIMCypA was PCR amplified from a pLHA-TRIMCypA-SN vector (27) and inserted into the pSicor vector downstream of T2A by using the XmaI and EcoRI restriction enzymes (New England Biolabs). To construct the HIV-1_NL4_._3_-Δenv-eGFP proviral plasmid, eGFP was inserted into the *env* open reading frame of WT or mutant HIV-1_NL4.3_ (59) with the BsaBI and NheI restriction enzymes (New England Biolabs).

### Site-directed mutagenesis.

QuikChange mutagenesis was used to make mutations in the pDEST-RTp51-syn-V5-6×His vector, the pFC32K-RTp66 NanoBRET vector, or the pGCHIV-1_NL4_._3_ shuttle vector (contains a partial HIV-1 sequence) with Phusion polymerase (New England Biolabs) and complementary primers with the required nucleotide changes. After mutagenesis of the pGSHIV-1_NL4_._3_ shuttle vector, the pGCHIV-1_NL4_._3_ shuttle vector and HIV-1_NL4.3_ were digested with EcoRI and SphI and the mutated shuttle fragment was ligated into digested HIV-1_NL4.3_ to construct HIV-1_NL4_._3_-pol-E297R, -E298R, and -E300R. Mutations in all plasmids were confirmed by sequencing.

### Co-IP and Western blotting.

Co-IP was performed as previously described (17, 18). Briefly, Dynabeads Protein G (Thermo, Fisher) were coupled to rabbit anti-eEF1A antibody (Cell Signaling Technology) overnight at 4°C. HEK293T cells expressing WT or mutant HIV-1 RT with V5 and 6×His tags were lysed in S100 buffer (10 mM Tris [pH 7.4], 1.5 mM MgCl_2_, 10 mM KCl, 1× cOmplete protease inhibitor cocktail [Roche], 0.5 mM β-mercaptoethanol) with a Dounce homogenizer. The cell lysate was cleared by centrifugation (20,000 × *g*, 10 min, 4°C), and 100 µl of lysate supernatant was incubated with eEF1A-coupled Dynabeads for 2 h at 4°C. The beads were separated with a magnet and washed three times for 20 min in S100 buffer plus 0.3% Triton X-100 and 300 mM NaCl. The beads were then boiled at 100°C for 10 min, and proteins were subjected to Western blot analysis with anti-V5, anti-RT, and anti-eEF1A antibodies (Santa Cruz) as indicated. Where indicated, Western blots were probed with anti-HIV-1 immunoglobulin (HIV-IG, obtained through the NIH AIDS Reagent Program, Division of AIDS, NIAID, NIH; catalog no. 3957, HIV-IG from NABI and NHLBI).

### NanoBRET.

NanoBRET assay was performed in accordance with the manufacturer’s instructions (Promega). Briefly, HEK293T cells were cotransfected in the wells of a 24-well plate with 5 ng of WT or mutant RTp66-NanoLuc (donor) and 500 ng of HaloTag-eEF1A (acceptor) for 6 h. Cells were trypsinized, and 40,000 cells were transferred to the wells of a 96-well white opaque plate with HaloTag NanoBRET 618 Ligand (Promega) and incubated overnight at 37°C. Nano-Glo substrate was added to each well, and the plate was read on a BioTek Synergy H4 plate reader with a 450-nm band-pass filter and a 610-nm long-pass filter to measure donor emission and ligand emission, respectively. The BRET ratio was calculated by dividing the 610-nm emission reading by the 450-nm emission reading, and this was used to calculate the BRET ratio of the mutant RTs relative to that of WT RT. The BRET ratio of the negative control (RSV phosphoprotein [P] cotransfected with HaloTag-eEF1A) relative to that of WT RT was subtracted from all samples, and then these were renormalized to WT RT.

### MAPPIT.

The interaction between the HIV-1 RTp51 and RTp66 subunits as a model of RT heterodimerization was performed with the MAPPIT system as described previously (18, 60). Briefly, HEK293T cells were transfected with 400 ng of RTp51-bait plasmid (WT or mutant), 500 ng of RTp66-prey (WT or mutant) plasmid, and 100 ng of reporter plasmid pXP2d2-rPAP1-luciferase. A bait construct with the myeloid differentiation primary response protein 88 (hMvD88) and a prey construct with simian virus 40 large T antigen (SVT) were used as negative controls. After 24 h of transfection, recombinant mouse leptin (R&D Systems, USA) was added to cells at 100 ng/ml and the mixture was incubated for a further 24 h before the cells were lysed in Glo Lysis Buffer (Promega) and firefly luciferase activity was measured in the cell lysate.

### Purification of WT and E300R mutant RTp51/p66 heterodimer from *E. coli*.

The RTp51/p66 heterodimer expression system, with a 6×His-tagged WT RTp51/p66 heterodimer expression plasmid (p6HRT-PROT) and the *lacI* expression plasmid (pDM1.1), and the original purification method used have been previously described (25). The E300R mutation was introduced into p6HRT-PROT by inverse PCR with Phusion polymerase (New England Biolabs), and the mutation was confirmed by sequencing. The primers used to generate p6HRT-PROT-E300R were as follows: forward, 5′-CGTCTAGAACTGGCAGAAAACAGA-3′; reverse, 5′-TGCTTCTTCTGTTAGTGGTATTACTT-3′. The WT or E300R mutant p6HRT-PROT expression vector and the pDM1.1 vector were cotransformed into *E. coli* BL21(DE3) (New England Biolabs). Transformants were cultured in Power Prime-Olate broth (Sapphire Bioscience) with ampicillin (100 μM) and kanamycin (50 μM) at 37°C. When the *E. coli* culture optical density at 600 nm reached 0.4 to 0.8, expression was induced with 400 µM isopropyl-β-d-thiogalactopyranoside (IPTG; Sigma-Aldrich). The cells were harvested after 5 h of induction, and the cell pellets were lysed in 1× FastBreak cell lysis reagent (Promega). The 6×His-tagged WT RTp51/p66 heterodimer was purified with MagneHis Ni-Particles (Promega). After binding with RT, the beads were washed in wash buffer (50 mM NaH_2_PO_4_/Na_2_HPO_4_, 300 mM NaCl, 20 mM imidazole, pH 7.8) six times. RT was then eluted in elution buffer (50 mM NaH_2_PO_4_-Na_2_HPO_4_, 300 mM NaCl, 200 mM imidazole, pH 6.5). RTp66 and RTp51 monomers were separated from RT heterodimers with an Ultra-0.5 Centrifugal Filter Unit with Ultracel-100 membrane (Amicon). The concentration of purified RT heterodimer was measured with the Bradford assay (Bio-Rad).

### BLI assay.

Purified recombinant eEF1A1 (OriGene) was biotinylated with EZ-Link-sulfo-NHS-biotin in accordance with the manufacturer’s instructions (Pierce Biotechnology, IL, USA) and immobilized on Octet Red system streptavidin-coated biosensors (Pall ForteBio, CA, USA). Biosensors with eEF1A1 bound were incubated in 90 nM WT or E300R RTp51/p66 heterodimer purified from *E. coli* to allow association (absorption rate constant [*K*_*a*_]) and then washed in a standard kinetic buffer (1 mM phosphate, 15 mM NaCl, 0.002% Tween 20, 0.1 mg/ml gelatini) to allow dissociation (dissociation constant [*K*_*d*_]). The binding affinity (*K*_*D*_) was calculated by dividing the *K*_*D*_ by the *K*_*a*_.

### ERT assay.

WT or E300R HIV-1 virions (~20 ng) were used in an ERT assay as previously described (61). Briefly, virions were incubated with 500 U/ml DNase I and 10 mM MgCl_2_ with or without 200 µM dNTPs for 6 h. DNA was extracted once in an equal volume of phenol-chloroform-isoamyl alcohol (25:24:1) and once in chloroform before ethanol precipitation and resuspension of precipitated DNA in 0.1 mM EDTA H_2_O. ssDNA and first strand transfer DNA were measured by qPCR as previously described (37), with iTaq Universal SYBR Green Supermix (Bio-Rad).

### Virus infection and analysis of reverse transcription by qPCR.

HeLa cells were seeded into six-well plates and incubated overnight to reach confluence the following day. VSV-G-pseudotyped WT or mutant HIV-1-_NL4.3_ was treated with 200 U/ml DNase I–5 mM MgCl_2_ for 1 h at 37°C to remove any HIV-1_NL4.3_ plasmid DNA prior to addition to cells, and where indicated, virus was heat inactivated at 100°C for 30 min. WT or mutant HIV-1 was added to HEK293T cells with 8 µg/ml Polybrene and spinoculated at 1,200 × *g* for 1.5 h at 16°C and then for 4 h at 37°C. Cells were washed three times with 0.1 mM EDTA phosphate-buffered saline (PBS) and then were lysed with Glo Lysis Buffer (Promega). The ssDNA, first strand transfer DNA, and late-stage DNA products of reverse transcription were measured by qPCR with iTaq Universal SYBR Green Supermix (Bio-Rad) as previously described (37).

### Nevirapine addition assay.

HeLa cells were seeded at 20,000/well of a 96-well plate overnight and infected with 5 ng of CA of VSV-G-pseudotyped HIV-1_NL4_._3_-Δenv-eGFP by spinoculation at 1,200 × *g* for 1.5 h at 16°C. Virus medium was removed, cells were washed with PBS, and the medium was replaced with warmed RPMI or warmed RPMI containing 5 µM nevirapine at the postinfection times indicated. Cells were incubated for 48 h at 37°C and 5% CO_2_ before fixation with 1% paraformaldehyde, and eGFP was analyzed by flow cytometry.

### Cyclosporine A washout assay.

The pSicor-mCherry-T2A-Pro-TRIMCypA lentivirus vector was transduced into HeLa cells, which were sorted for mCherry-positive cells, termed HeLa-TC cells (HeLa TRIMCypA-expressing cells). The CsA washout assay was performed as previously described (5, 14, 27). Briefly, 20,000 sorted HeLa-TC cells were seeded into the wells of 96-well plates and incubated overnight. The following day, the medium was replaced with RPMI containing 2.5 µM CsA (Merck Millipore) and 8 µg/ml Polybrene. The CsA binds to CypA in the TRIMCypA fusion protein, which prevents its binding to HIV-1 CA and restricting infection; thus, infection continued unimpeded. Ten nanograms of CA of VSV-G-pseudotyped HIV-1_NL4_._3_-Δenv-eGFP was added to the wells, and the plates were spinoculated at 1,200 × *g* for 1.5 h at 16°C. Virus medium was removed, cells were washed with PBS, and fresh medium containing 2.5 µM CsA or the equivalent volume of 100% ethanol was added. The medium was replaced with nonsupplemented cell culture medium at the postinfection times indicated, cells were incubated for 48 h at 37°C in 5% CO_2_ before fixation with 1% paraformaldehyde, and eGFP and mCherry levels were analyzed by flow cytometry.

### Statistical analysis.

Statistical analyses were performed with an ordinary one-way analysis of variance (ANOVA) with multiple comparisons comparing each column to the WT (Fig. 3B, 4A, 5A and C, and 6A and C). Repeated-measures one-way ANOVA with multiple comparisons comparing each column to the WT was used for the *in vitro* RT catalytic activity assay, where slight differences in the HIV-1 stock p24 value can significantly affect the results of the sensitive assay (Fig. 4C). An ordinary two-way ANOVA comparing each column (HIV-1 CA) within each row (time point) was conducted for the HIV-1 replication kinetics (Fig. 7). An unpaired Student *t* test was performed for the endogenous reverse transcription (ERT) assays in Fig. 4D to F. The statistical significance of differences between data is indicated as follows: *, *P* < 0.05; **, *P* < 0.01; ***, *P* < 0.001; ****, *P* < 0.0001.
