# Supplementary material for: HIV-1 Uncoating and Reverse Transcription Require eEF1A Binding to Surface-Exposed Acidic Residues of the Reverse Transcriptase Thumb Domain
Source: mBio. 2018 Mar 27;9(2):e00316-18. doi: 10.1128/mBio.00316-18 (PMC5874916; doi:10.1128/mBio.00316-18)
Supplement: FIG S1 [file mbo002183799sf1.pdf]

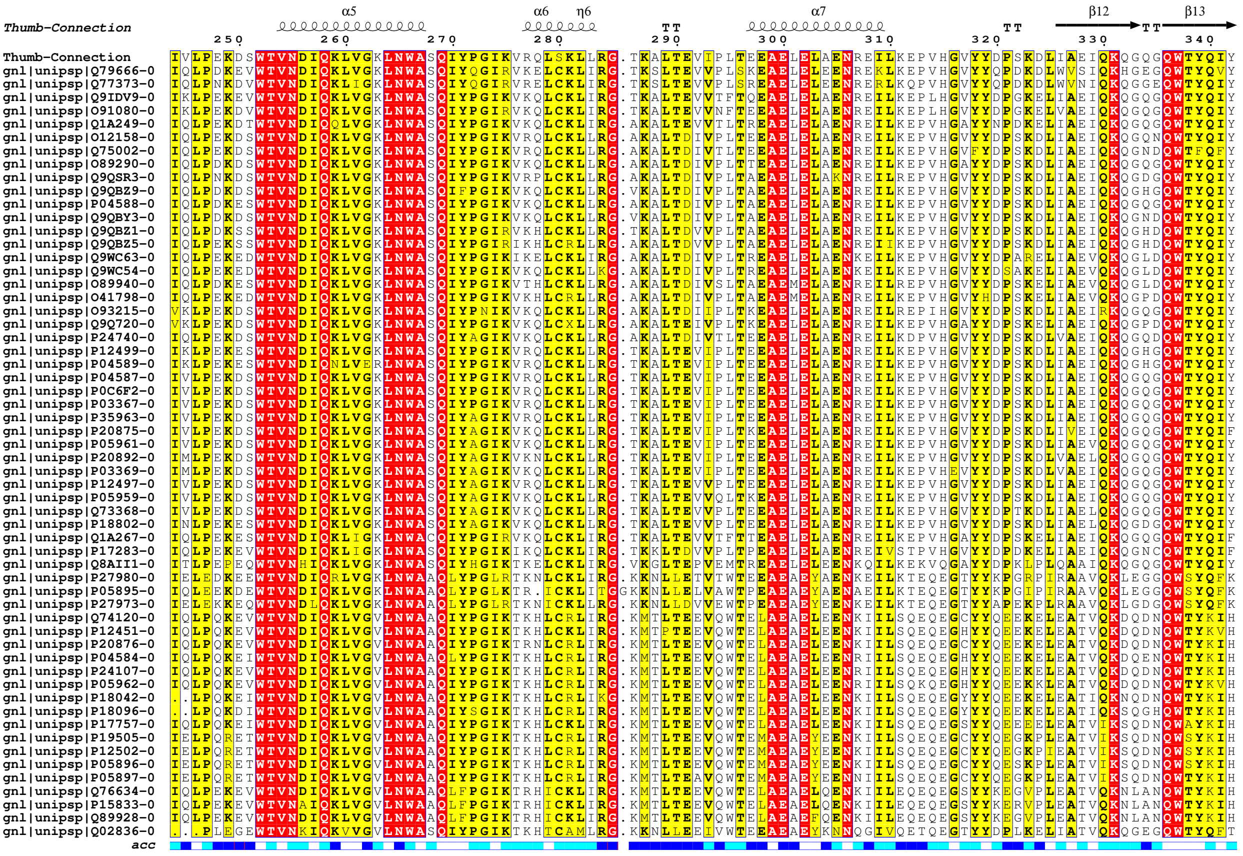
Thumb-Connection
α5
α6
η6
TT
α7
TT
β12
TT
β13
250
260
270
280
290
300
310
320
330
340
Thumb-Connection IVLPEKDSWTVNDIQKLVGKLNWASQIYPGIKVRQLSKLLRG.TKALTEVIPLTEEAELELAENREILKEPVHGVYYDPSKDLIAEIQKQGQGQWTYQIY
gnl|unipsp|Q79666-0 IQLPDKEVWTVNDIQKLVGKLNWASQIYQGIRVKELCKLIRG.TKSLTEVVPLSKEAELELEENREKLKEPVHGVYYQPDKDLWVSIQKHGEGQWTYQVY
gnl|unipsp|Q77373-0 IQLPNKDVWTVNDIQKLIGKLNWASQIYQGIRVRELCKLIRG.TKSLTEVVPLSREAELELEENRERLKQPVHGVYYQPDKDLWVNIQKQGGEQWTYQIY
gnl|unipsp|Q9IDV9-0 IKLPEKEVWTVNDIQKLVGKLNWASQIYPGIKVKQLCKLIRG.TKALTEVVTFTQEAELELAENREILKEPLHGVYYDPGKELIAEIQKQGQGQWTYQIY
gnl|unipsp|O91080-0 IKLPEKDVWTVNDIQKLVGKLNWASQIYPGIRVKQLCKLIRG.TKALTEVVNFTEEAELELAENREILKEPLHGVYYDPGKELVAEIQKQGQGQWTYQIY
gnl|unipsp|Q1A249-0 IQLPEKDTWTVNDIQQLVGKLNWASQIYPGIKVKQLCKLIRG.AKALTEVVTLTREAELELAENREILKEPVHGAYYNPDKELIAEIQKQGQGQWTYQIY
gnl|unipsp|O12158-0 IQLPEKDSWTVNDIQKLVGKLNWASQIYPGIKVRQLCKLLRG.AKALTDIVTLTEEAELELAENREILKEPVHGVYYDPSKDLIAEIQKQGQNQWTYQIY
gnl|unipsp|Q75002-0 IQLPEKDSWTVNDIQKLVGKLNWASQIYPGIKVRQLCKLLRG.AKALTDIVTLTEEAELELAENREILKEPVHGVFYDPSKDLIAEIQKQGNDQWTFQFY
gnl|unipsp|O89290-0 IQLPDKDSWTVNDIQKLVGKLNWASQIYPGIKVKQLCKLLRG.AKALTDIVPLTTEAELELAENREILKEPVHGAYYDPSKDLIAEIQKQGQGQWTYQIY
gnl|unipsp|Q9QSR3-0 IQLPNKDSWTVNDIQKLVGKLNWASQIYPGIKVRPLCKLLRG.AKALTDIVPLTAEAELELAKNREILREPVHGVYYDPSKDLIAEIQKQGDGQWTYQIY
gnl|unipsp|Q9QBZ9-0 IQLPDKDSWTVNDIQKLVGKLNWASQIFPGIKVKQLCKLLRG.VKALTDIVPLTAEAELELAENREILKEPVHGVYYDPSKDLIAEIQKQGHGQWTYQIY
gnl|unipsp|P04588-0 IQLPDKESWTVNDIQKLVGKLNWASQIYPGIKVKQLCKLLRG.AKALTDIVPLTAEAELELAENREILKEPVHGVYYDPSKDLIAEIQKQGQGQWTYQIY
gnl|unipsp|Q9QBY3-0 IQLPDKDSWTVNDIQKLVGKLNWASQIYPGIKVKQLCKLLRG.VKALTDIVPLTAEAELELAENREILKEPVHGVYYDPSKDLIAEIQKQGNDQWTYQIY
gnl|unipsp|Q9QBZ1-0 IQLPDKSSWTVNDIQKLVGKLNWASQIYPGIRVKHLCKLLRG.TKALTDVVPLTAEAELELAENREILKEPVHGVYYDPSKDLIAEIQKQGHDQWTYQIY
gnl|unipsp|Q9QBZ5-0 IQLPEKSSWTVNDIQKLVGKLNWASQIYPGIRIKHLCRLLRG.AKALTDVVPLTAEAELELAENREIIKEPVHGVYYDPSKDLIAEIQKQGHDQWTYQIY
gnl|unipsp|Q9WC63-0 IQLPEKEDWTVNDIQKLVGKLNWASQIYPGIKIKELCKLIRG.AKALTDIVPLTREAELELAENKEILKEPVHGVYYDPARELIAEVQKQGLDQWTYQIY
gnl|unipsp|Q9WC54-0 IQLPEKEDWTVNDIQKLVGKLNWASQIYPGIKVKQLCKLLKG.AKALTDIVPLTREAELELAENKEILKEPVHGVYYDSAKELIAEVQKQGLDQWTYQIY
gnl|unipsp|O89940-0 IQLPDKESWTVNDIQKLVGKLNWASQIYPGIKVTHLCKLLKG.AKALTDIVSLTAEAEMELAENREILREPVHGVYYDPSKELIAEVQKQGLDQWTYQIY
gnl|unipsp|O41798-0 IQLPEKEDWTVNDIQKLVGKLNWASQIYPGIKVKHLCRLLRG.AKALTDIVPLTAEAEMELAENREILKEPVHGVYHDPSKELIAEVQKQGPDQWTYQIY
gnl|unipsp|O93215-0 VKLPEKDSWTVNDIQKLVGKLNWASQIYPNIKVKQLCKLLRG.AKALTDIIPLTKEAELELAENREILREPIHGVYYDPSKDLIAEIRKQGQGQWTYQIY
gnl|unipsp|Q9Q720-0 VKLPEKDSWTVNDIQKLVGKLNWASQIYPGIKVKQLCXLLRG.AKALTEIVPLTKEAELELAENREILKEPVHGAYYDPSKELIAEIQKQGPDQWTYQIY
gnl|unipsp|P24740-0 IQLPEKDSWTVNDIQKLVGKLNWASQIYAGIKVKQLCKLLRG.AKALTDIVTLTEEAELELAENREILKDPVHGVYYDPSKDLVAEIQKQGQDQWTYQIY
gnl|unipsp|P12499-0 IKLPEKESWTVNDIQKLVGKLNWASQIYPGIKVRQLCKLLRG.TKALTEVIPLTEEAELELAENREILKEPVHGVYYDPSKDLIAEIQKQGHGQWTYQIY
gnl|unipsp|P04589-0 IKLPEKESWTVNDIQNLVERLNWASQIYPGIKVRQLCKLLRG.TKALTEVIPLTEEAELELAENREILKEPVHGVYYDPSKDLIAEIQKQGHGQWTYQIY
gnl|unipsp|P04587-0 IVLPEKDSWTVNDIQKLVGKLNWASQIYPGIKVRQLCKLLRG.TKALTEVIPLTEEAELELAENREILKEPVHGVYYDPSKDLIAEIQKQGQGQWTYQIY
gnl|unipsp|P0C6F2-0 IVLPEKDSWTVNDIQKLVGKLNWASQIYPGIKVRQLCKLLRG.TKALTEVIPLTEEAELELAENREILKEPVHGVYYDPSKDLIAEIQKQGQGQWTYQIY
gnl|unipsp|P03367-0 IVLPEKDSWTVNDIQKLVGKLNWASQIYPGIKVRQLCKLLRG.TKALTEVIPLTEEAELELAENREILKEPVHGVYYDPSKDLIAEIQKQGQGQWTYQIY
gnl|unipsp|P35963-0 IVLPEKDSWTVNDIQKLVGKLNWASQIYAGIKVRQLCKLLRG.TKALTEVIPLTEEAELELAENREILKEPVHGVYYDPSKDLIAEIQKQGQGQWTYQIY
gnl|unipsp|P20875-0 IVLPEKDSWTVNDIQKLVGKLNWASQIYAGIKVKQLCKLLRG.TKALTEVIPLTKEAELELAENREILKEPVHGVYYDPSKDLIVEIQKQGQGQWTYQIF
gnl|unipsp|P05961-0 IVLPEKDSWTVNDIQKLVGKLNWASQIYAGIKVKQLCKLLRG.TKALTEVIPLTEEAELELAENREILKEPVHGVYYDPSKDLIAEVQKQGQGQWTYQIY
gnl|unipsp|P20892-0 IMLPEKDSWTVNDIQKLVGKLNWASQIYAGIKVKNLCKLLRG.TKALTEVIPLTEEAELELAENREILKEPVHGVYYDPSKDLVAELQKQGQGQWTYQIY
gnl|unipsp|P03369-0 IMLPEKDSWTVNDIQKLVGKLNWASQIYAGIKVKQLCKLLRG.TKALTEVIPLTEEAELELAENREILKEPVHEVYYDPSKDLVAEIQKQGQGQWTYQIY
gnl|unipsp|P12497-0 IVLPEKDSWTVNDIQKLVGKLNWASQIYAGIKVRQLCKLLRG.TKALTEVVPLTEEAELELAENREILKEPVHGVYYDPSKDLIAEIQKQGQGQWTYQIY
gnl|unipsp|P05959-0 IVLPEKDSWTVNDIQKLVGKLNWASQIYAGIKVKQLCKLLRG.TKALTEVVQLTKEAELELAENREILKEPVHGVYYDPSKDLIAEIQKQGQGQWTYQIY
gnl|unipsp|Q73368-0 IVLPEKDSWTVNDIQKLVGKLNWASQIYAGIKVKQLCKLLRG.TKALTEVVPLTEEAELELAENREILKEPVHGVYYDPTKDLIAELQKQGQGQWTYQIY
gnl|unipsp|P18802-0 INLPEKESWTVNDIQKLVGKLNWASQIYAGIKVKQLCKLLRG.TKALTEVVPLTEEAELELAENREILKEPVHGVYYDPSKDLIAELQKQGDGQWTYQIY
gnl|unipsp|Q1A267-0 IQLPEKESWTVNDIQKLIGKLNWACQIYPGIRVKQLCKLIRG.TKALTEVVTFTTEAELELAENREILKEPVHGAYYDPSKELIAEIQKQGQGQWTYQIF
gnl|unipsp|P17283-0 IQLPEKEVWTVNDIQKLIGKLNWASQIYPGIKIKQLCKLIRG.TKKLTDVVPLTPEAELELAENREIVSTPVHGVYYDPDKELIAEIQKQGNCQWTYQIF
gnl|unipsp|Q8AII1-0 ITLPEPEQWTVNHIQKLVGKLNWASQIYHGIKTKELCKLIRG.VKGLTEPVEMTREAELELEENKQILKEKVQGAYYDPKLPLQAAIQKQGQGQWTYQIY
gnl|unipsp|P27980-0 IELEDKEEWTVNDIQRLVGKLNWAAQLYPGLRTKNLCKLIRG.KKNLLETVTWTEEAEAEYAENKEILKTEQEGTYYKPGRPIRAAVQKLEGGQWSYQFK
gnl|unipsp|P05895-0 IQLEEKDEWTVNDIQKLVGKLNWAAQLYPGLKTR.ICKLITGGKKNLLELVAWTPEAEAEYAENAEILKTEQEGTYYKPGIPIRAAVQKLEGGQWSYQFK
gnl|unipsp|P27973-0 IELEKKEQWTVNDLQKLVGKLNWAAQLYPGLRTKNICKLLRG.KKNLLDVVEWTPEAEAEYEENKEILKTEQEGTYYAPEKPLRAAVQKLGDGQWSYQFK
gnl|unipsp|Q74120-0 IQLPQKEVWTVNDIQKLVGVLNWAAQIYPGIKTKHLCRLIRG.KMTLTEEVQWTELAEAELEENKIILSQEQEGCYYQEEKELEATVQKDQDNQWTYKIH
gnl|unipsp|P12451-0 IQLPQKEVWTVNDIQKLVGVLNWAAQIYPGIKTKHLCKLIRG.KMTPTEEVQWTELAEAELEENKIILSQEQEGHYYQEEKELEATVQKDQDNQWTYKVH
gnl|unipsp|P20876-0 IQLPQKEVWTVNDIQKLVGVLNWAAQIYPGIKTRNLCRLIRG.KMTLTEEVQWTELAEAELEENKIILSQEQEGCYYQEEKELEATVQKDQDNQWTYKIH
gnl|unipsp|P04584-0 IQLPQKEIWTVNDIQKLVGVLNWAAQLYPGIKTKHLCRLIRG.KMTLTEEVQWTELAEAELEENRIILSQEQEGHYYQEEKELEATVQKDQENQWTYKIH
gnl|unipsp|P24107-0 IQLPQKEVWTVNDIQKLVGVLNWAAQIYPGIKTKHLCRLIRG.KMTLTEEVQWTELAEAELEENRIILSQEQEGHYYQEEKELEATVQKDQDNQWTYKIH
gnl|unipsp|P05962-0 IQLPQKEVWTVNDIQKLVGVLNWAAQIYPGIKTKHLCRLIRG.KMTLTEEVQWTELAEAELEENRIILSQKQEGHYYQEEKKLEATVQKDQDNQWTYKVH
gnl|unipsp|P18042-0 ..LPQKEIWTVNDIQKLVGVLNWAAQIYPGIKTKHLCRLIKG.KMTLTEEVQWTELAEAELEENKIILSQEQEGYYYQEEKELEATIQKNQDNQWTYKIH
gnl|unipsp|P18096-0 ..LPQKDIWTVNDIQKLVGVLNWAAQIYSGIKTKHLCRLIRG.KMTLTEEVQWTELAEAELEENKIILSQEQEGYYYQEEKELEATIQKSQGHQWTYKIH
gnl|unipsp|P17757-0 IQLPQKEIWTVNDIQKLVGVLNWAAQIYPGIKTKHLCKLIRG.KMTLTEEVQWTELAEAELEENKIILSQEQEGSYYQEEEELEATVIKSQDNQWAYKIH
gnl|unipsp|P19505-0 IELPQRETWTVNDIQKLVGVLNWAAQIYPGIKTKHLCRLIRG.KMTLTEEVQWTEMAEAEYEENKIILSQEQEGCYYQEGKPLEATVIKSQDNQWSYKIH
gnl|unipsp|P12502-0 IELPQRETWTVNDIQKLVGVLNWAAQIYPGIKTKHLCRLIRG.KMTLTEEVQWTEMAEAEYEENKIILSQEQEGCYYQEGKPIEATVIKSQDNQWSYKIH
gnl|unipsp|P05896-0 IELPQRETWTVNDIQKLVGVLNWAAQIYPGIKTKHLCRLIRG.KMTLTEEVQWTEMAEAEYEENKIILSQEQEGCYYQESKPLEATVIKSQDNQWSYKIH
gnl|unipsp|P05897-0 IELPQRETWTVNDIQKLVGVLNWAAQIYPGIKTKHLCRLIRG.KMTLTEAVQWTEMAEAEYEENNIILSQEQEGCYYQEGKPLEATVIKSQDNQWTYKIH
gnl|unipsp|Q76634-0 IQLPEKEVWTVNDIQKLVGVLNWAAQLFPGIKTRHICKLIRG.KMTLTEEVQWTELAEAELQENKIILEQEQEGSYYKEGVPLEATVQKNLANQWTYKIH
gnl|unipsp|P15833-0 IQLPEKEVWTVNAIQKLVGVLNWAAQLFPGIKTRHICKLIRG.KMTLTEEVQWTELAEAELQENKIILEQEQEGSYYKERVPLEATVQKNLANQWTYKIH
gnl|unipsp|Q89928-0 IQLPEKEVWTVNDIQKLVGVLNWAAQLFPGIKTRHICKLIRG.KMTLTEEVQWTELAEAEFQENKIILEQEQEGSYYKEGVPLEATVQKNLANQWTYKIH
gnl|unipsp|Q02836-0 ...PLEGEWTVNKIQKVVGVLNWASQIYPGIKTKHTCAMLRG.KKNLLEEIVWTEEAEAEYKNNQGIVQETQEGTYYDPLKELIATVQKQGEGQWTYQFT
acc
