# Supplementary material for: HIV-1 Uncoating and Reverse Transcription Require eEF1A Binding to Surface-Exposed Acidic Residues of the Reverse Transcriptase Thumb Domain
Source: mBio. 2018 Mar 27;9(2):e00316-18. doi: 10.1128/mBio.00316-18 (PMC5874916; doi:10.1128/mBio.00316-18)
Supplement: TABLE S1 [file mbo002183799st1.docx]

| **Supporting Table S1: Hydrogen bonding frequency residues in the thumb domain of the wild-type reverse transcriptase subunit p66 and mutants during molecular dynamics simulations. Mutated residues are shown in red and the color code used for the frequency is shown at the bottom of the table.** | | | | | | | | | | | | | | | | | | | | |  |
| --- | --- | --- | --- | --- | --- | --- | --- | --- | --- | --- | --- | --- | --- | --- | --- | --- | --- | --- | --- | --- | --- |
|  | **Wild-Type** | | | **W252A** | | | **L303A** | | | | | **E298R** | | | | | **E300R** | | | |  |
| **Residue** | **Donor** | **Acceptor** | **%** | **Donor** | **Acceptor** | **%** | **Donor** | | **Acceptor** | | **%** | **Donor** | | **Acceptor** | **%** | | **donor** | **Acceptor** | | **%** |  |
| **248** | GLU248-Main | GLU248-Side | **5** | GLU248-Main | GLU248-Side | **15** | GLU248-Main | | GLU248-Side | | **5** | GLU248-Main | | GLU248-Side | **6** | | GLU248-Main | GLU248-Side | | **18** |  |
|  | ARG307-Side | GLU248-Side | **17** | ARG307-Side | GLU248-Side | **2** | ARG307-Side | | GLU248-Side | | **21** | ARG307-Side | | GLU248-Side | **28** | | ARG307-Side | GLU248-Side | | **44** |  |
| **249** | LYS249-Side | ASP256-Side | **17** | LYS249-Side | ASP256-Side | **14** | LYS249-Side | | ASP256-Side | | **64** | LYS249-Side | | ASP256-Side | **6** | | LYS249-Side | ASP256-Side | | **34** |  |
|  | TRP252-Side | LYS249-Main | **16** | ALA252-Side | LYS249-Main | **0** | TRP252-Side | | LYS249-Main | | **0** | TRP252-Side | | LYS249-Main | **2** | | TRP252-Side | LYS249-Main | | **7** |  |
| **250** | ASP250-Main | ASP250-Side | **3** | ASP250-Main | ASP250-Side | **2** | ASP250-Main | | ASP250-Side | | **2** | ASP250-Main | | ASP250-Side | **11** | | ASP250-Main | ASP250-Side | | **0** |  |
|  | LEU295-Main | ASP250-Main | **15** | LEU295-Main | ASP250-Main | **43** | LEU295-Main | | ASP250-Main | | **2** | LEU295-Main | | ASP250-Main | **0** | | LEU295-Main | ASP250-Main | | **56** |  |
|  | GLU300-Side | ASP250-Side | **0** | GLU300-Side | ASP250-Side | **0** | GLU300-Side | | ASP250-Side | | **0** | GLU300-Side | | ASP250-Side | **0** | | ARG300-Side | ASP250-Side | | **59** |  |
| **252** | TRP252-Side | PRO247-Main | **0** | ALA252-Side | PRO247-Main | **0** | TRP252-Side | | PRO247-Main | | **4** | TRP252-Side | | PRO247-Main | **11** | | TRP252-Side | PRO247-Main | | **0** |  |
|  | TRP252-Side | LYS249-Main | **16** | ALA252-Side | LYS249-Main | **0** | TRP252-Side | | LYS249-Main | | **0** | TRP252-Side | | LYS249-Main | **2** | | TRP252-Side | LYS249-Main | | **7** |  |
|  | TRP252-Main | ILE293-Main | **67** | ALA252-Main | ILE293-Main | **8** | TRP252-Main | | ILE293-Main | | **5** | TRP252-Main | | ILE293-Main | **65** | | TRP252-Main | ILE293-Main | | **20** |  |
| **253** | THR253-Main | ASP256-Side | **79** | THR253-Main | ASP256-Side | **70** | THR253-Main | | ASP256-Side | | **71** | THR253-Main | | ASP256-Side | **87** | | THR253-Main | ASP256-Side | | **46** |  |
|  | THR253-Side | ASP256-Side | **81** | THR253-Side | ASP256-Side | **35** | THR253-Side | | ASP256-Side | | **23** | THR253-Side | | ASP256-Side | **96** | | THR253-Side | ASP256-Side | | **35** |  |
|  | ASP256-Main | THR253-Side | **23** | ASP256-Main | THR253-Side | **18** | ASP256-Main | | THR253-Side | | **12** | ASP256-Main | | THR253-Side | **25** | | ASP256-Main | THR253-Side | | **15** |  |
| **254** | GLN258-Main | VAL254-Main | **28** | GLN258-Main | VAL254-Main | **52** | GLN258-Main | | VAL254-Main | | **52** | GLN258-Main | | VAL254-Main | **17** | | GLN258-Main | VAL254-Main | | **49** |  |
|  | VAL254-Main | GLU291-Main | **15** | VAL254-Main | GLU291-Main | **17** | VAL254-Main | | GLU291-Main | | **16** | VAL254-Main | | GLU291-Main | **27** | | VAL254-Main | GLU291-Main | | **5** |  |
| **297** | GLU298-Side | GLU297-Side | **0** | GLU298-Side | GLU297-Side | **0** | GLU298-Side | | GLU297-Side | | **0** | ARG298-Side | | GLU297-Side | **64** | | GLU298-Side | GLU297-Side | | **0** |  |
|  | LEU301-Main | GLU297-Main | **6** | LEU301-Main | GLU297-Main | **16** | LEU301-Main | | GLU297-Main | | **21** | LEU301-Main | | GLU297-Main | **6** | | LEU301-Main | GLU297-Main | | **11** |  |
| **298** | LYS281-Side | GLU298-Side | **27** | LYS281-Side | GLU298-Side | **6** | LYS281-Side | | GLU298-Side | | **5** | LYS281-Side | | ARG298-Side | **0** | | LYS281-Side | GLU298-Side | | **8** |  |
|  | GLU302-Main | GLU298-Main | **0** | GLU302-Main | GLU298-Main | **26** | GLU302-Main | | GLU298-Main | | **35** | GLU302-Main | | ARG298-Main | **0** | | GLU302-Main | GLU298-Main | | **13** |  |
| **299** | ALA299-Main | THR296-Side | **1** | ALA299-Main | THR296-Side | **23** | ALA299-Main | | THR296-Side | | **15** | ALA299-Main | | THR296-Side | **1** | | ALA299-Main | THR296-Side | | **11** |  |
|  | LEU303-Main | ALA299-Main | **13** | VAL303-Main | ALA299-Main | **39** | ALA303-Main | | ALA299-Main | | **47** | LEU303-Main | | ALA299-Main | **6** | | LEU303-Main | ALA299-Main | | **28** |  |
| **300** | GLU300-Side | GLU248-Side | **0** | GLU300-Side | GLU248-Side | **0** | GLU300-Side | | GLU248-Side | | **0** | GLU300-Side | | GLU248-Side | **0** | | ARG300-Side | GLU248-Side | | **44** |  |
|  | GLU300-Side | ASP250-Side | **0** | GLU300-Side | ASP250-Side | **0** | GLU300-Side | | ASP250-Side | | **0** | GLU300-Side | | ASP250-Side | **0** | | ARG300-Side | ASP250-Side | | **59** |  |
|  | GLU300-Main | THR296-Main | **16** | GLU300-Main | THR296-Main | **25** | GLU300-Main | | THR296-Main | | **31** | GLU300-Main | | THR296-Main | **15** | | ARG300-Main | THR296-Main | | **20** |  |
|  | ALA304-Main | GLU300-Main | **14** | ALA304-Main | GLU300-Main | **19** | ALA304-Main | | GLU300-Main | | **21** | ALA304-Main | | GLU300-Main | **14** | | ALA304-Main | ARG300-Main | | **14** |  |
| **302** | LYS275-Side | GLU302-Side | **15** | LYS275-Side | GLU302-Side | **20** | LYS275-Side | | GLU302-Side | | **49** | LYS275-Side | | GLU302-Side | **1** | | LYS275-Side | GLU302-Side | | **18** |  |
|  | ARG277-Main | GLU302-Side | **37** | ARG277-Main | GLU302-Side | **1** | ARG277-Main | | GLU302-Side | | **0** | ARG277-Main | | GLU302-Side | **8** | | ARG277-Main | GLU302-Side | | **0** |  |
|  | ARG277-Side | GLU302-Side | **38** | ARG277-Side | GLU302-Side | **43** | ARG277-Side | | GLU302-Side | | **64** | ARG277-Side | | GLU302-Side | **0** | | ARG277-Side | GLU302-Side | | **92** |  |
|  | GLN278-Main | GLU302-Side | **56** | GLN278-Main | GLU302-Side | **21** | GLN278-Main | | GLU302-Side | | **2** | GLN278-Main | | GLU302-Side | **88** | | GLN278-Main | GLU302-Side | | **4** |  |
|  | GLN278-Side | GLU302-Side | **11** | GLN278-Side | GLU302-Side | **3** | GLN278-Side | | GLU302-Side | | **23** | GLN278-Side | | GLU302-Side | **13** | | GLN278-Side | GLU302-Side | | **17** |  |
|  | LEU279-Main | GLU302-Side | **37** | LEU279-Main | GLU302-Side | **5** | LEU279-Main | | GLU302-Side | | **0** | LEU279-Main | | GLU302-Side | **55** | | LEU279-Main | GLU302-Side | | **1** |  |
|  | GLU302-Main | GLU298-Main | **2** | GLU302-Main | GLU298-Main | **26** | GLU302-Main | | GLU298-Main | | **35** | GLU302-Main | | ARG298-Main | **0** | | GLU302-Main | GLU298-Main | | **13** |  |
|  | ASN306-Side | GLU302-Main | **14** | ASN306-Side | GLU302-Main | **0** | ASN306-Side | | GLU302-Main | | **0** | ASN306-Side | | GLU302-Main | **4** | | ASN306-Side | GLU302-Main | | **0** |  |
|  | ASN306-Main | GLU302-Main | **4** | ASN306-Main | GLU302-Main | **32** | ASN306-Main | | GLU302-Main | | **48** | ASN306-Main | | GLU302-Main | **0** | | ASN306-Main | GLU302-Main | | **24** |  |
| **303** | LEU303-Main | ALA299-Main | **13** | LEU303-Main | ALA299-Main | **39** | ALA303-Main | | ALA299-Main | | **47** | LEU303-Main | | ALA299-Main | **6** | | LEU303-Main | ALA299-Main | | **28** |  |
|  | ARG307-Main | LEU303-Main | **13** | ARG307-Main | LEU303-Main | **29** | ARG307-Main | | ALA303-Main | | **34** | ARG307-Main | | LEU303-Main | **7** | | ARG307-Main | LEU303-Main | | **24** |  |
| **305** | LYS275-Side | GLU305-Side | **46** | LYS275-Side | GLU305-Side | **31** | LYS275-Side | | GLU305-Side | | **19** | LYS275-Side | | GLU305-Side | **89** | | LYS275-Side | GLU305-Side | | **28** |  |
|  | ARG277-Side | GLU305-Side | **38** | ARG277-Side | GLU305-Side | **0** | ARG277-Side | | GLU305-Side | | **0** | ARG277-Side | | GLU305-Side | **0** | | ARG277-Side | GLU305-Side | | **0** |  |
|  | GLU305-Main | LEU301-Main | **3** | GLU305-Main | LEU301-Main | **17** | GLU305-Main | | LEU301-Main | | **22** | GLU305-Main | | LEU301-Main | **2** | | GLU305-Main | LEU301-Main | | **9** |  |
|  | ILE309-Main | GLU305-Main | **1** | ILE309-Main | GLU305-Main | **11** | ILE309-Main | | GLU305-Main | | **13** | ILE309-Main | | GLU305-Main | **1** | | ILE309-Main | GLU305-Main | | **4** |  |
| **307** | ARG307-Side | LEU246-Main | **7** | ARG307-Side | LEU246-Main | **22** | ARG307-Side | | LEU246-Main | | **4** | ARG307-Side | | LEU246-Main | **0** | | ARG307-Side | LEU246-Main | | **37** |  |
|  | ARG307-Side | PRO247-Main | **8** | ARG307-Side | PRO247-Main | **29** | ARG307-Side | | PRO247-Main | | **4** | ARG307-Side | | PRO247-Main | **0** | | ARG307-Side | PRO247-Main | | **20** |  |
|  | ARG307-Side | GLU248-Side | **17** | ARG307-Side | GLU248-Side | **2** | ARG307-Side | | GLU248-Side | | **21** | ARG307-Side | | GLU248-Side | **2** | | ARG307-Side | GLU248-Side | | **18** |  |
|  | ARG307-Main | LEU303-Main | **13** | ARG307-Main | LEU303-Main | **29** | ARG307-Main | | ALA303-Main | | **34** | ARG307-Main | | LEU303-Main | **7** | | ARG307-Main | LEU303-Main | | **24** |  |
|  | ARG307-Side | GLU308-Side | **8** | ARG307-Side | GLU308-Side | **6** | ARG307-Side | | GLU308-Side | | **8** | ARG307-Side | | GLU308-Side | **34** | | ARG307-Side | GLU308-Side | | **6** |  |
| **308** | GLU308-Main | ALA304-Main | **5** | GLU308-Main | ALA304-Main | **8** | GLU308-Main | | ALA304-Main | | **15** | GLU308-Main | | ALA304-Main | **4** | | GLU308-Main | ALA304-Main | | **6** |  |
|  | ARG307-Side | GLU308-Side | **8** | ARG307-Side | GLU308-Side | **6** | ARG307-Side | | GLU308-Side | | **8** | ARG307-Side | | GLU308-Side | **34** | | ARG307-Side | GLU308-Side | | **6** |  |
|  | LYS311-Side | GLU308-Side | **2** | LYS311-Side | GLU308-Side | **14** | LYS311-Side | | GLU308-Side | | **18** | LYS311-Side | | GLU308-Side | **2** | | LYS311-Side | GLU308-Side | | **9** |  |
| **311** | LYS311-Side | GLU308-Side | **2** | LYS311-Side | GLU308-Side | **14** | LYS311-Side | | GLU308-Side | | **18** | LYS311-Side | | GLU308-Side | **2** | | LYS311-Side | GLU308-Side | | **0** |  |
| **312** | TYR271-Side | GLU312-Main | **49** | TYR271-Side | GLU312-Main | **8** | TYR271-Side | | GLU312-Main | | **5** | TYR271-Side | | GLU312-Main | **50** | | TYR271-Side | GLU312-Main | | **16** |  |
|  | LYS353-Side | GLU312-Side | **15** | LYS353-Side | GLU312-Side | **1** | LYS353-Side | | GLU312-Side | | **12** | LYS353-Side | | GLU312-Side | **0** | | LYS353-Side | GLU312-Side | | **0** |  |
| **Absolute change in hydrogen bond frequency compared to wild-type p66** | | | | | | | | **0.1-0.19** | | **0.20-0.29** | | | **0.30-0.39** | | | **0.4-0.49** | | | **>0.5** | | |
| **Absolute increase** | | | | | | | |  | |  | | |  | | |  | | |  | | |
| **Absolute decrease** | | | | | | | |  | |  | | |  | | |  | | |  | | |
