# Supplementary figures and images for: HIV-1 Uncoating and Reverse Transcription Require eEF1A Binding to Surface-Exposed Acidic Residues of the Reverse Transcriptase Thumb Domain
Source: mBio. 2018 Mar 27;9(2):e00316-18. doi: 10.1128/mBio.00316-18 (PMC5874916; doi:10.1128/mBio.00316-18)

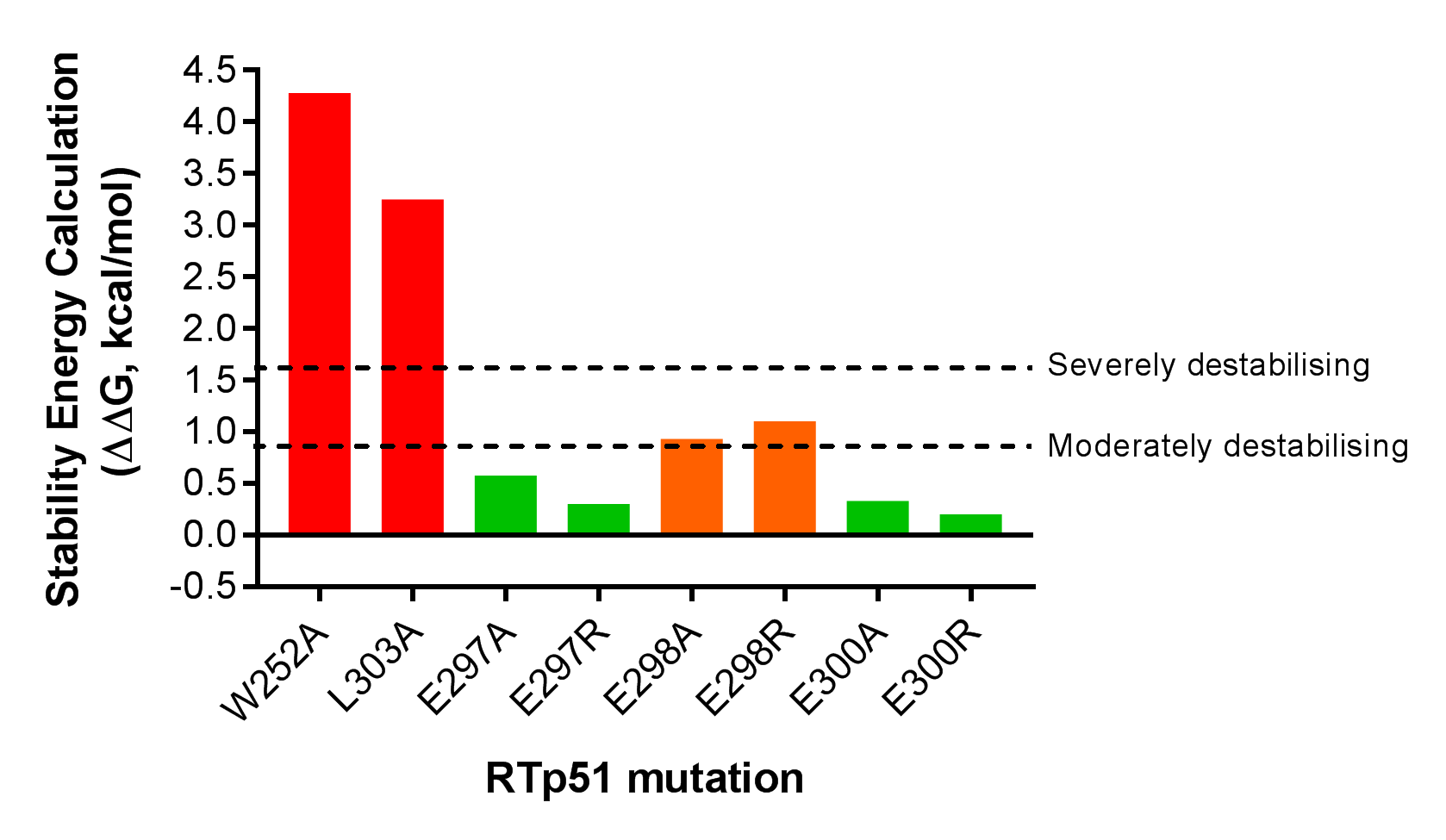

Supplement: FIG S2 [file mbo002183799sf2.tif]

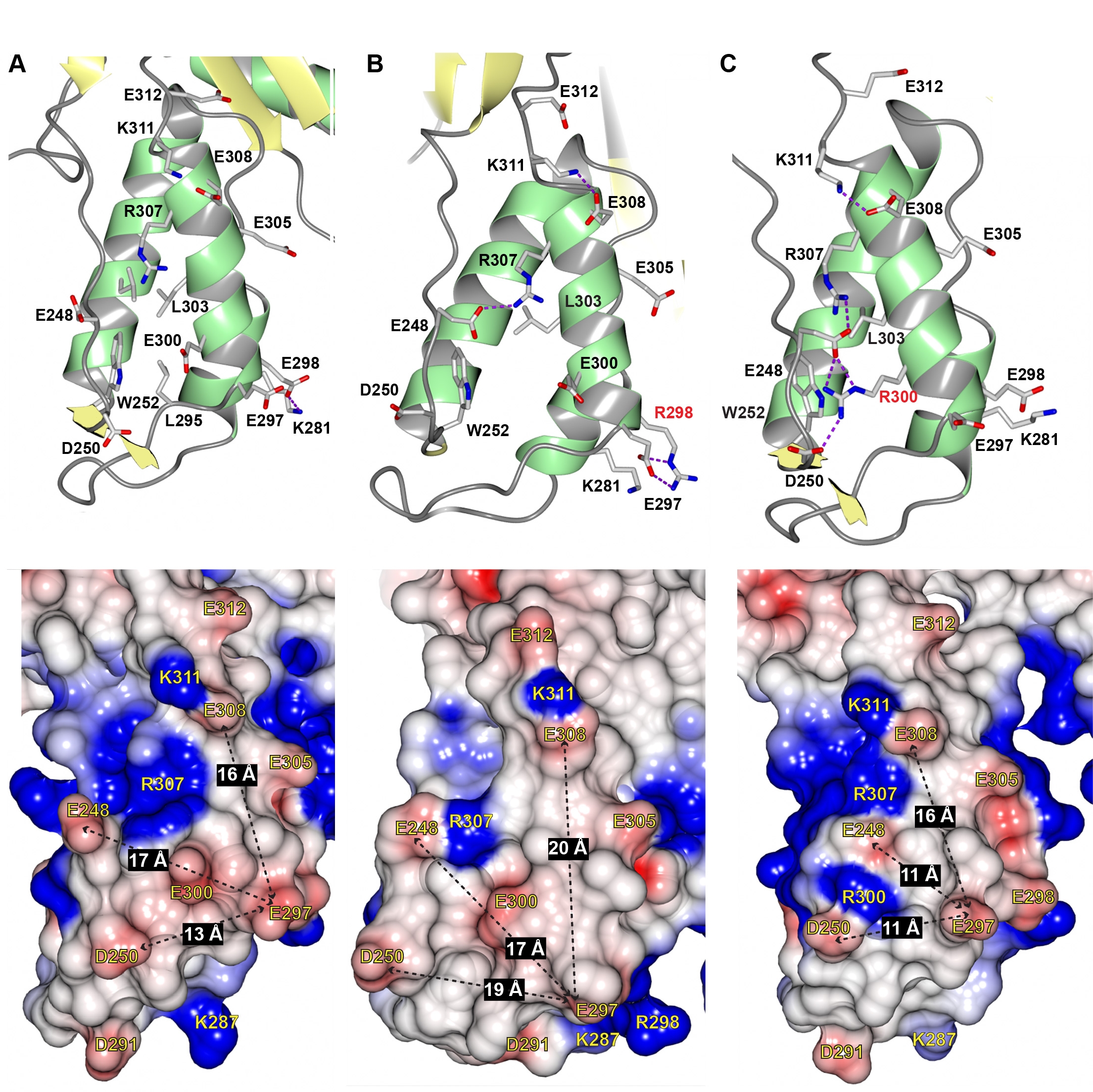

Supplement: FIG S3 [file mbo002183799sf3.jpg]

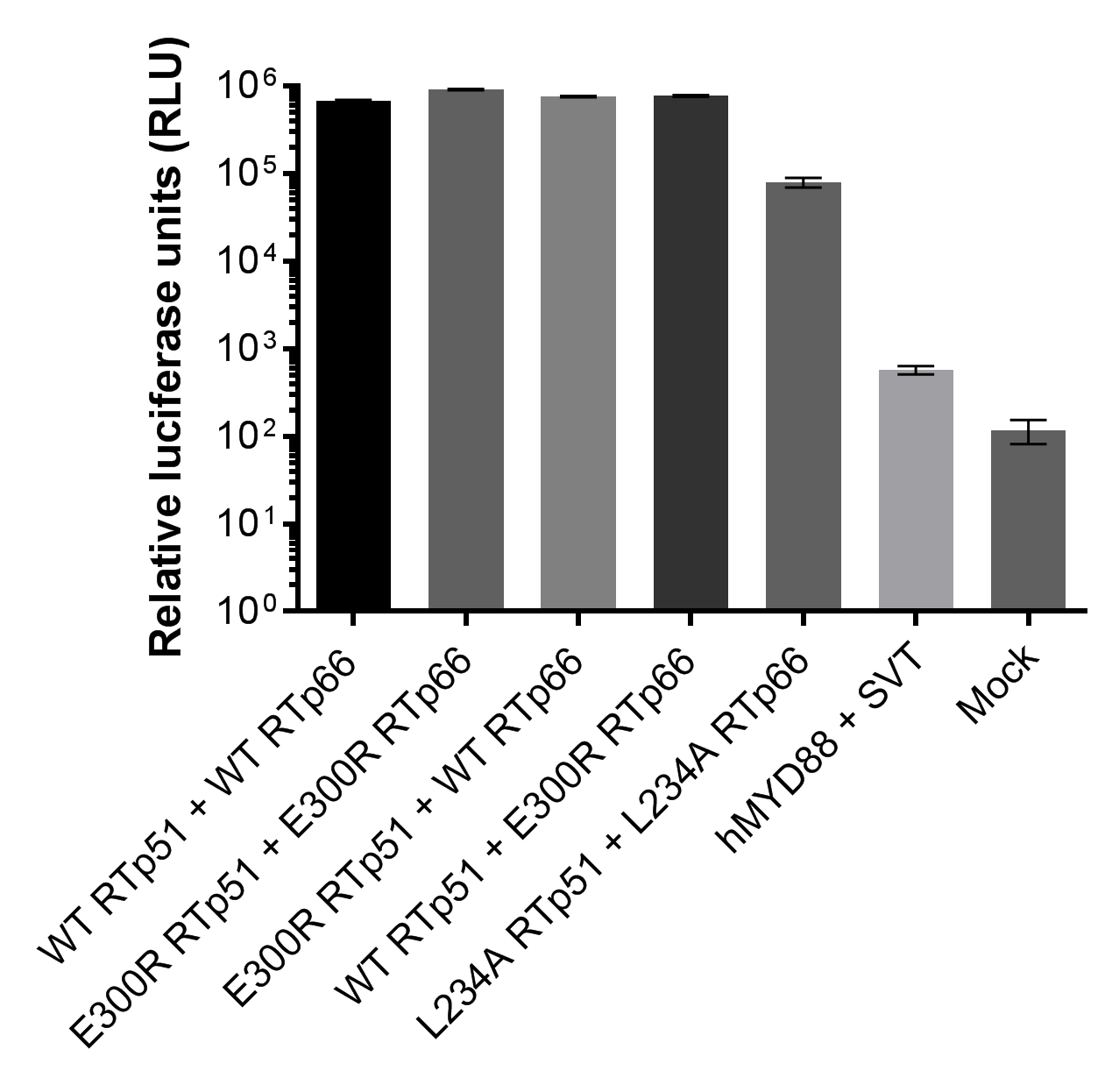

Supplement: FIG S5 [file mbo002183799sf5.tif]

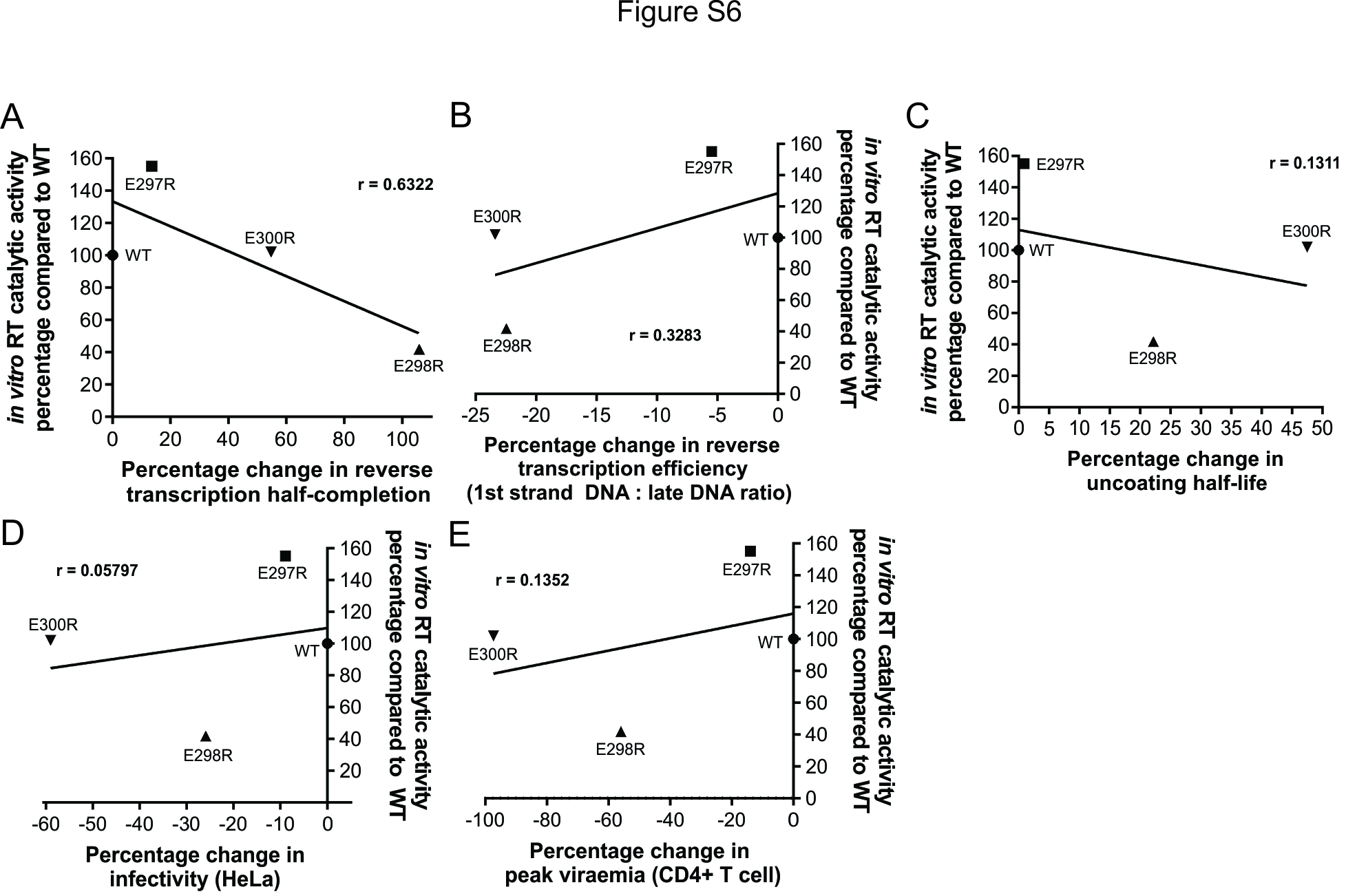

Supplement: FIG S6 [file mbo002183799sf6.tif]
